# Engineering *Tripterygium wilfordii*-derived exosome-like nanoparticles for targeted therapy in rheumatoid arthritis

**DOI:** 10.20517/evcna.2026.66

**Published:** 2026-09-21

**Authors:** Yunyi Yang, Zhi Gong, Gongming Shi, Yong Yang, Yun He, Hongjie Yang, Dajian Yang, Xiaohong Yang

**Affiliations:** ^1^Yueyang Hospital of Integrated Traditional Chinese and Western Medicine, Shanghai University of Traditional Chinese Medicine, Shanghai 200437, China.; ^2^Translational Innovation Center, Shenzhen Bay Laboratory, Shenzhen 518132, Guangdong, China.; ^3^Chongqing Institute of Medicinal Plant Cultivation, Chongqing 408435, China.; ^4^Chongqing Academy of Chinese Materia Medica, Chongqing 400065, China.; ^5^Chongqing Institute of Green and Intelligent Technology, Chinese Academy of Sciences, Chongqing 400714, China.; ^#^These authors contributed equally to this work.

**Keywords:** Rheumatoid arthritis, exosome-like nanoparticles, *Tripterygium wilfordii*

## Abstract

**Aim:** Rheumatoid arthritis (RA) is a chronic autoimmune disease characterized by excessive activation of pro-inflammatory macrophages and elevated levels of reactive oxygen species (ROS), resulting in synovial hyperplasia, cartilage destruction, and joint dysfunction. This study aimed to develop an engineered *Tripterygium wilfordii*-derived exosome-like nanoparticle system (TWELP@GlcN-HA) that integrates antioxidant, anti-inflammatory, immunomodulatory, and joint-targeting activities for synergistic RA therapy.

**Methods:***Tripterygium wilfordii*-derived exosome-like nanoparticles (TWELP) were isolated from fresh *Tripterygium wilfordii* and sequentially surface-modified with glucosamine (GlcN) for cartilage protection and hyaluronic acid (HA) for targeted uptake by inflammatory cells. Their physicochemical properties, including morphology, particle size, structural integrity, and surface potential, were systematically characterized. *In vitro* assays were performed to evaluate ROS scavenging, inhibition of pro-inflammatory cytokines [tumor necrosis factor-α (TNF-α) and interleukin-6 (IL-6)], and macrophage polarization. Biodistribution and therapeutic efficacy were evaluated in a collagen-induced arthritis (CIA) mouse model by assessing synovial inflammation, cartilage damage, joint morphology, and joint function.

**Results:** TWELP@GlcN-HA exhibited a uniform spherical morphology, stable surface potential, and preserved structural integrity. *In vitro*, it efficiently scavenged ROS, suppressed TNF-α and IL-6 secretion, and promoted macrophage polarization from M1 to M2, thereby restoring immune homeostasis. *In vivo*, HA-mediated targeting promoted nanoparticle accumulation in inflamed joints. TWELP@GlcN-HA significantly alleviated synovial inflammation and cartilage destruction, improved joint morphology and function, and exhibited excellent biosafety with no systemic toxicity.

**Conclusion:** TWELP@GlcN-HA provides a multifunctional combinatorial strategy for RA treatment by synergistically regulating ROS, reprogramming pro-inflammatory macrophages, protecting cartilage, and achieving targeted joint delivery. Its enhanced therapeutic efficacy and favorable safety profile make it a promising candidate for future clinical translation in RA management.

## INTRODUCTION

Rheumatoid arthritis (RA) is a systemic autoimmune disease characterized by chronic synovial inflammation and progressive joint destruction. Its increasing global incidence, high rate of disability, and substantial socioeconomic burden pose major public health challenges^[1-3]^. The pathogenesis of RA involves genetic susceptibility, environmental factors, and immune dysregulation^[4,5]^. Aberrant activation of autoreactive T cells induces fibroblast-like synoviocyte (FLS) proliferation, matrix metalloproteinase (MMP) secretion, and receptor activator of nuclear factor-κB ligand (RANKL) upregulation, thereby promoting cartilage degradation and bone erosion^[6-8]^. Persistent RA progression is closely associated with an imbalance in the local inflammatory microenvironment. M1 macrophages produce high levels of reactive oxygen species (ROS) and pro-inflammatory cytokines, such as tumor necrosis factor-α (TNF-α) and interleukin-1β (IL-1β), which drive synovial hyperplasia, cartilage destruction, and bone erosion. Meanwhile, an insufficient number or activity of anti-inflammatory M2 macrophages impedes effective resolution of inflammation^[9,10]^. Excessive ROS production further activates the NOD-, LRR- and pyrin domain-containing protein 3 (NLRP3) inflammasome, amplifying inflammatory responses and reinforcing the M1-dominant state^[11-13]^. Current treatments, including nonsteroidal anti-inflammatory drugs, disease-modifying antirheumatic drugs, and biological agents, alleviate symptoms but do not fully disrupt this inflammatory-oxidative cycle. Moreover, their long-term use may cause adverse effects such as interstitial lung disease, bone marrow suppression, hyperglycemia, hepatotoxicity, and infection^[14-17]^. Strategies that simultaneously scavenge ROS and promote M1-to-M2 macrophage polarization have therefore emerged as promising approaches to restoring immune homeostasis in affected joints^[18,19]^.

*Tripterygium wilfordii*, a traditional antirheumatic medicinal plant, exerts anti-inflammatory, immunomodulatory, antiproliferative, and bone-protective effects. However, its clinical application is limited by its poor solubility, narrow therapeutic window, and multiorgan toxicity^[20,21]^. Plant-derived exosome-like nanoparticles (PDENs) have recently emerged as promising delivery platforms because of their natural origin, low immunogenicity, and ability to cross biological barriers^[22]^. PDENs are enriched in lipids, proteins, and plant-derived small molecules and can scavenge ROS and induce macrophage polarization, providing a natural platform for treating immune-related diseases^[23,24]^.

In this study, we developed an engineered *Tripterygium wilfordii*-derived exosome-like nanoparticle system (TWELP@GlcN-HA) that synergistically regulates ROS and pro-inflammatory macrophages within the RA microenvironment. *Tripterygium wilfordii*-derived exosome-like nanoparticles (TWELP) were isolated from fresh *Tripterygium wilfordii* and functionalized with glucosamine (GlcN) to protect cartilage and DSPE-PEG_2000_-hyaluronic acid (HA) to facilitate targeted uptake by inflammatory cells. TWELP@GlcN-HA combines antioxidant, anti-inflammatory, immunomodulatory, and joint-protective activities, effectively alleviating synovial inflammation and osteochondral destruction. This multifunctional nanoparticle-based strategy offers a novel approach to targeted RA treatment.

## METHODS

### Materials

Unless otherwise stated, all reagents and solvents were obtained from commercial suppliers and used without further purification. RAW264.7 murine macrophages, human fibroblast-like synoviocytes (HFLS and HFLS-RA), and the MH7A human rheumatoid synovial cell line were acquired from the Center for Excellence in Molecular Cell Science, Chinese Academy of Sciences (Shanghai, China). Cell authentication was performed by the cell bank. All cells were routinely tested for mycoplasma contamination using the Myco-Lumi^TM^ Luminescent Mycoplasma Detection Kit (Beyotime, Shanghai, China) and confirmed mycoplasma-negative before use. Cells at passage 3 (P3; passage range P2-P4) were used throughout the experiments. Female BALB/c mice [6-8 weeks old, specific pathogen-free (SPF)] were supplied by Chongqing Byrness Weil Biotechnology Co., Ltd. (Chongqing, China). All animal experiments were approved by the Experimental Animal Welfare and Ethics Review Committee of Chongqing Academy of Chinese Materia Medica (Approval No. CACMMEAC20250021) and were conducted in accordance with the approved institutional guidelines.

### Isolation of TWELP

Fresh *T. wilfordii* plants were washed and homogenized in phosphate-buffered saline (PBS) (pH 7.4). The homogenate was sequentially centrifuged at 1,000 × *g* for 10 min, 3,000 × *g* for 20 min, and 10,000 × *g* for 40 min at 4 °C to remove debris and large particles. The supernatant was further purified using an exosome extraction column (EVscore05; Aidimai Biotechnology, Nanjing, China), and fractions containing exosome-like nanoparticles were collected. TWELP were resuspended in PBS and stored at -80 °C until use.

### Synthesis of DBCO-GlcN and DSPE-PEG_2000_-HA

As illustrated in Supplementary Figure 1, dibenzocyclooctyne (DBCO)-GlcN and DSPE-PEG_2000_-HA were synthesized as functional conjugates for TWELP surface modification, providing cartilage-protective and HA-targeting capabilities. DBCO-GlcN was prepared through a multi-step sequence involving *O*-alkylation, amidation, and Schiff base formation with *D*-glucosamine, and its structure was confirmed by ^1^H NMR, ^13^C NMR, and high-resolution mass spectrometry (HRMS) [Supplementary Figure 2]. DSPE-PEG_2000_-HA was obtained by coupling HA with DSPE-PEG_2000_-NH_2_ via carbodiimide chemistry and characterized by ^1^H NMR [Supplementary Figure 3]. Detailed procedures are provided in the Supplementary Materials.

### Preparation of TWELP@GlcN-HA

For bioorthogonal conjugation, TWELP (1 mg/mL, based on total protein) was first reacted with N_3_-PEG_4_-NHS (10 mM; Energy Chemical, China) in PBS (pH 7.4) at 4 °C for 2 h to introduce azide groups. Unreacted reagents were removed by ultrafiltration [molecular weight cut-off (MWCO) 100 kDa] to obtain TWELP-N_3_. TWELP-N_3_ was then incubated with DBCO-GlcN (1 mM) at 4 °C for 2 h to yield GlcN-modified TWELP (TWELP@GlcN). Subsequently, TWELP@GlcN was incubated with DSPE-PEG_2000_-HA (1 mg/mL) at room temperature for 1 h to anchor the polymer onto the vesicle membrane. The final TWELP@GlcN-HA was purified by ultrafiltration (MWCO 100 kDa) to remove unbound components. The TWELP@HA was prepared using the same HA-insertion procedure but without the azide-introduction and DBCO-GlcN-conjugation steps. Detailed proportions and preparation conditions are provided in the Supplementary Materials.

### Characterization of TWELP, TWELP@GlcN and TWELP@GlcN-HA

The morphology of TWELP, TWELP@GlcN and TWELP@GlcN-HA was observed by transmission electron microscopy (TEM, Hitachi HT7700, Japan) operated at 100 kV. Particle size distribution, concentration, and ζ-potential were determined using a nanoparticle tracking analysis system (NTA, ZetaView PMX 120, Particle Metrix, Germany). Protein profiles of TWELP before and after surface modification were analyzed by 12% sodium dodecyl sulfate-polyacrylamide gel electrophoresis (SDS-PAGE) followed by Coomassie Brilliant Blue staining to evaluate preservation of the protein profile. Total protein loading for SDS-PAGE was quantified by bicinchoninic acid (BCA) assay before electrophoresis. The successful conjugation of DBCO-GlcN to azide-modified TWELP (TWELP-N_3_) was indirectly verified via a DBCO-Cy5.5-based fluorescent labeling assay and competitive fluorescent labeling assay, with detailed experimental procedures provided in the Supplementary Materials. The loading efficiencies of GlcN and HA within TWELP@GlcN-HA were quantified using high-performance liquid chromatography (HPLC) and gel permeation high-performance liquid chromatography (GPC-HPLC), respectively.

### Cellular uptake of TWELP@GlcN-HA

Cellular uptake of fluorescently labeled TWELP and TWELP@GlcN-HA by RAW264.7 macrophages was investigated according to a previously reported method with minor modifications^[25]^. TWELP were labeled using the exosome-specific fluorescent staining kit FluoExo® Exosome Tracing Staining Kit-Green (Echo Biotech, China) according to the manufacturer’s instructions and previous reports^[26]^. After labeling, excess unbound fluorescent dye was removed using an exosome-exclusive purification column. RAW264.7 cells were seeded in 24-well plates (1 × 10^4^ cells/well) and cultured for 24 h with or without lipopolysaccharide (LPS) stimulation (100 ng/mL; Sigma-Aldrich, USA). The medium was then replaced with fresh medium containing fluorescently labeled TWELP or TWELP@GlcN-HA, and the cells were incubated for 4 h. To investigate the contribution of HA-cluster of differentiation 44 (CD44) interactions to cellular uptake, cells were pretreated with excess free HA (500 μg/mL) for 1 h as a competitive blocking ligand to occupy HA-binding sites on CD44 before exposure to TWELP@GlcN-HA. After incubation, cells were washed three times with PBS, fixed with 4% paraformaldehyde for 20 min, and counterstained with 4’,6-diamidino-2-phenylindole (DAPI). Nanoparticle internalization was visualized by green fluorescence using confocal laser scanning microscopy (CLSM, Leica TCS SP8, Germany).

### In vitro anti-inflammatory and antioxidant assays

The anti-inflammatory and antioxidant activities of TWELP@GlcN-HA were evaluated in RAW264.7 macrophages. For the anti-inflammatory assay, cells (3 × 10^5^ cells/well) were seeded in 24-well plates and cultured for 12 h. The normal Control group was maintained without LPS stimulation and treated with PBS. Cells in all other groups were stimulated with LPS (500 ng/mL) for 12 h to induce an inflammatory phenotype. The medium was then replaced with fresh medium containing PBS (LPS group), triptolide (TP) (10 μg/mL), GlcN (50 μg/mL), TWELP (50 μg/mL), TWELP@HA (50 μg/mL), or TWELP@GlcN-HA (50 μg/mL), and the cells were incubated for an additional 12 h. Supernatants were collected, and IL-6, TNF-α, and IL-1β levels were quantified by enzyme-linked immunosorbent assay (ELISA).

For the antioxidant assay, cells (1 × 10^4^ cells/well) were seeded in 24-well plates and cultured for 24 h. Cells were pretreated for 2 h with PBS (Control and LPS groups), TP (10 μg/mL), GlcN (50 μg/mL), TWELP (50 μg/mL), TWELP@HA (50 μg/mL), or TWELP@GlcN-HA (50 μg/mL). All groups except the normal Control group were then stimulated with LPS (500 ng/mL) for 4 h to induce oxidative stress. Intracellular ROS were detected using a ROS detection kit (Beyotime, Shanghai, China) according to the manufacturer’s instructions, and fluorescence images were acquired using an inverted fluorescence microscope after 30 min of staining.

### Live/dead staining

HFLS-RA and MH7A cells (5 × 10^4^/well) were seeded in 12-well plates for 24 h and treated with PBS (control), TP (10 μg/mL), GlcN (50 μg/mL), TWELP (50 μg/mL), TWELP@HA (50 μg/mL), or TWELP@GlcN-HA (50 μg/mL) for 24 h. Cells were then washed and stained with Calcein-AM/PI (30 min, 37 °C, dark) following the kit instructions, washed again, and imaged using an inverted fluorescence microscope (Leica DMi8, Germany). Live cells fluoresced green, and dead cells red; representative images were acquired from randomly selected fields for qualitative assessment.

### In vitro macrophage polarization analysis

RAW264.7 cells were seeded on glass coverslips in 24-well plates and cultured for 12 h. M1 polarization was induced with LPS (500 ng/mL, 12 h), and M2 polarization with interleukin-4 (IL-4) (20 ng/mL, 12 h). After stimulation, cells were treated with PBS (control), TP (10 μg/mL), GlcN (50 μg/mL), TWELP (50 μg/mL), TWELP@HA (50 μg/mL), or TWELP@GlcN-HA (50 μg/mL) for 12 h. Cells were fixed with 4% paraformaldehyde (20 min), washed with PBS, and blocked with 5% goat serum (1 h, room temperature). For M1 analysis, cells were incubated with anti-CD86 (1:200) overnight at 4 °C, followed by fluorescent secondary antibody (1:500, 1 h, room temperature); nuclei were counterstained with DAPI (10 min). M2 analysis was performed similarly using anti-CD206. Fluorescence images were acquired by CLSM (Leica TCS SP8).

### RNA-sequencing and metabolomics

RAW264.7 cells were seeded in 6-cm dishes, allow to attached overnight, and stimulated with LPS (500 ng/mL, 1 h), followed by treatment with PBS, TWELP (50 μg/mL), or TWELP@GlcN-HA (50 μg/mL) for 24 h (two biological replicates per group). Cells were washed, lysed in TRIzol (1 mL per 5 × 10^6^ cells), homogenized, snap-frozen, and stored at -80 °C. Total RNA was extracted, quality-checked (Agilent 2100 Bioanalyzer), and used for library preparation. Sequencing was performed on an Illumina NovaSeq 6000. Differential gene expression analysis was performed using DESeq2 based on raw read counts. Statistical significance was assessed using the Wald test, and the resulting *P* values were adjusted for multiple comparisons using the Benjamini-Hochberg procedure to control the false discovery rate (FDR). Genes with an absolute log_2_ fold change (|log_2_FC|) > 1 and an adjusted *P* value (FDR) < 0.05 were considered differentially expressed genes (DEGs). Gene Ontology (GO) and Kyoto Encyclopedia of Genes and Genomes (KEGG) enrichment analyses of the identified DEGs were performed using Fisher’s exact test, with *P* values adjusted using the Benjamini-Hochberg procedure. Metabolomic profiling was conducted using liquid chromatography-tandem mass spectrometry (LC-MS/MS) (Thermo Q Exactive Orbitrap) in positive- and negative-ion modes, and the resulting data were processed using Compound Discoverer 3.1.

### *In vivo* therapeutic evaluation in CIA mice model

Female BALB/c mice (6-8 weeks old, SPF) were used to establish a collagen-induced arthritis (CIA) model with minor modifications^[27]^. After 1 week of acclimatization, type II bovine collagen (2 mg/mL in 0.05 M acetic acid; Chondrex, USA) was emulsified 1:1 with complete Freund’s adjuvant (CFA, Sigma, USA). On day 0, mice received a 0.1 mL subcutaneous injection at the tail base for primary immunization, followed by a booster of 0.1 mL collagen emulsified with incomplete Freund’s adjuvant (IFA) on day 5. Following booster immunization, early clinical signs including paw swelling and erythema were observed. On day 6, one day after booster immunization, mice exhibiting early signs of arthritis were randomly allocated to the treatment groups using clinical arthritis scores for stratification, and treatment was initiated on the same day as an early therapeutic intervention. Mice received the indicated formulations via tail-vein injection every other day. The doses were as follows: TP and GlcN at 2 mg/kg; TWELP and TWELP@HA at 2 mg/kg TWELP equivalent (based on total protein); and TWELP@GlcN-HA at 2 mg/kg TWELP equivalent combined with 2 mg/kg GlcN. The Normal and Model groups received an equivalent volume of PBS vehicle according to the same administration schedule. On day 23, mice were sacrificed for blood and tissue collection for subsequent analyses.

### *In vivo* biodistribution

To evaluate the *in vivo* distribution of TWELP@GlcN-HA, formulations were labeled with the near-infrared dye DiR (1,1’-dioctadecyl-3,3,3’,3’-tetramethylindotricarbocyanine iodide; Invitrogen, USA). CIA mice were randomly assigned to three groups: Free DiR, DiR-TWELP, and DiR-TWELP@GlcN-HA, and injected via the tail vein with equivalent DiR doses (0.01 mg/mouse). Fluorescence images were acquired at 24, 48, and 72 h post-injection using an *In Vivo* Imaging System (IVIS) imaging system (PerkinElmer). At 72 h, mice were euthanized, and ankle joints were excised for *ex vivo* imaging and quantitative analysis. Mean fluorescence intensity (counts/s/pixel) was calculated as total counts divided by exposure time and the region area.

### Evaluation of anti-arthritic efficacy

Body weight, paw thickness, and arthritis scores (0-4 scale^[28]^) were monitored throughout the experiment. On day 23, mice were euthanized and ankle joints were harvested for micro-computed tomography (micro-CT) (Skyscan 1176, Bruker, Belgium) to assess bone destruction and microarchitecture, including bone volume (BV), bone volume fraction (BV/TV), bone mineral density (BMD), trabecular bone surface (Tb.BS), trabecular thickness (Tb.Th), and trabecular cavity CT value (Tb.Cav.CT_Value). Blood was collected for serum TNF-α, IL-1β, IL-6, osteoprotegerin (OPG), and RANKL measurements by ELISA, and the OPG/RANKL ratio was calculated to evaluate bone metabolism. Ankle joints were fixed, decalcified, paraffin-embedded, sectioned, and hematoxylin and eosin (H&E) stained to assess synovial hyperplasia and inflammation. Histological scoring of synovitis and cartilage erosion was performed using the histological scoring system (HSS).

### *In vivo* macrophage polarization

For *in situ* analysis of macrophage polarization, joint sections were subjected to immunofluorescence staining using anti-CD86 and anti-CD206 antibodies (1:200, 4 °C overnight), followed by incubation with fluorescent secondary antibodies (1:500) and DAPI nuclear staining. CLSM (Leica TCS SP8) was used to visualize and quantify M1 (CD86^+^) and M2 (CD206^+^) macrophage populations.

### Biosafety evaluation

The *in vitro* cytotoxicity of different formulations toward RAW264.7 and HFLS cells was evaluated using the 3-(4,5-dimethylthiazol-2-yl)-2,5-diphenyltetrazolium bromide (MTT) assay. Cells were seeded into 96-well plates at a density of 5 × 10^3^ cells per well and cultured in complete medium for 24 h to allow attachment. Subsequently, cells were treated with Vehicle (control), TP (0, 1, 2.5, 5, 10, 20, 30, 40, and 50 μg/mL), TWELP, and TWELP@GlcN-HA at various concentrations (0, 5, 12.5, 25, 50, 100, 150, 200, and 250 μg/mL) for 24 h. After treatment, 20 μL of MTT solution (5 mg/mL) was added to each well, and the plates were incubated at 37 °C for an additional 4 h. The culture medium was then carefully removed, and 150 μL of dimethyl sulfoxide (DMSO) was added to each well to dissolve the formazan crystals, followed by gentle shaking for 10 min. The absorbance was measured at 570 nm using a microplate reader, and cell viability was expressed relative to the Vehicle-treated control group. Each condition was tested in at least triplicate wells, and all experiments were performed independently three times.

For *in vivo* biosafety evaluation, blood samples were collected after treatment, and routine hematological parameters and liver/kidney function indices were measured. Major organs (heart, liver, spleen, lung and kidney) were harvested, fixed, and subjected to H&E staining to assess potential histopathological changes and systemic toxicity of the formulations^[29]^.

### Statistical analysis

All data are presented as the mean ± standard deviation (SD). Statistical analyses were performed using GraphPad Prism 9.0. Differences among multiple groups were analyzed using one-way analysis of variance (ANOVA) followed by appropriate multiple comparison tests. Time-dependent changes in body weight, clinical arthritis scores, and paw thickness were analyzed using two-way repeated-measures ANOVA, with measurements from the same animal matched across time, followed by Sidak’s multiple comparisons test. A two-tailed *P* value < 0.05 was considered statistically significant.

## RESULTS

### Isolation and characterization of TWELP

TWELP were isolated from fresh *Tripterygium wilfordii* tissues via gradient centrifugation and column purification. The overall preparation strategy and surface modification process for TWELP@GlcN-HA are illustrated in Figure 1A. TEM showed spherical vesicles with intact membranes [Figure 1B]. NTA revealed a narrow size distribution with an average diameter of 110.2 ± 4.6 nm [Figure 1C], and the ζ-potential in PBS (pH 7.4) was -15.25 ± 0.32 mV [Figure 1D]. SDS-PAGE indicated multiple proteins [Figure 1E]. These results confirm successful isolation of intact TWELP with uniform size and stable surface charge, suitable for surface modification and therapeutic applications.

**Figure 1:**
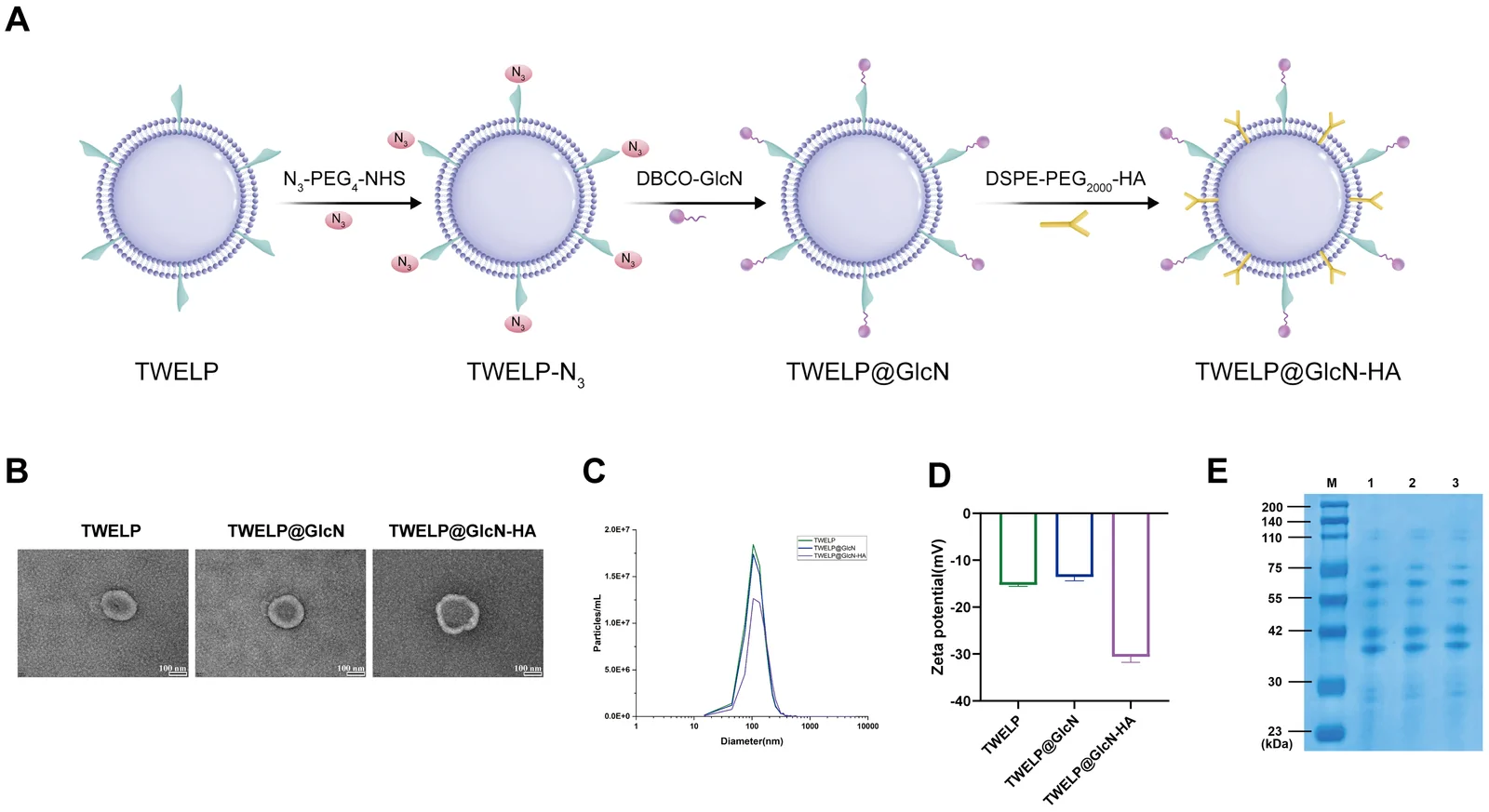
Preparation and characterization of TWELP@GlcN-HA. (A) Schematic of TWELP surface modification to generate TWELP@GlcN-HA; (B) TEM images of TWELP, TWELP@GlcN, and TWELP@GlcN-HA (scale bar = 100 nm); (C) NTA particle size distribution; (D) Zeta potential measured by NTA; (E) SDS-PAGE protein profiles (M: marker; lanes 1-3: TWELP, TWELP@GlcN, TWELP@GlcN-HA). DBCO-GlcN: Dibenzocyclooctyne-conjugated glucosamine; DSPE-PEG2000-HA: 1,2-distearoyl-sn-glycero-3-phosphoethanolamine-poly(ethylene glycol) 2000-hyaluronic acid conjugate; GlcN: glucosamine; HA: hyaluronic acid; N3-PEG4-NHS: azide-poly(ethylene glycol)4-N-hydroxysuccinimide ester; NTA: nanoparticle tracking analysis; SDS-PAGE: sodium dodecyl sulfate-polyacrylamide gel electrophoresis; TEM: transmission electron microscopy; TWELP: *Tripterygium wilfordii*-derived exosome-like nanoparticles; TWELP-N3: azide-modified TWELP; TWELP@GlcN: glucosamine-modified TWELP; TWELP@GlcN-HA: engineered *Tripterygium wilfordii*-derived exosome-like nanoparticle system.

### Characterization of TWELP@GlcN-HA

TWELP was sequentially functionalized with GlcN and HA to construct TWELP@GlcN-HA with cartilage-protective activity and HA-mediated targeting capability. GlcN was conjugated to TWELP via copper-free strain-promoted azide–alkyne cycloaddition (SPAAC) to endow the nanovesicles with cartilage-protective capacity, after which DSPE-PEG_2000_-HA was anchored onto the vesicle membrane through hydrophobic interactions for targeted delivery to inflamed synovial tissues.

TEM observations demonstrated that TWELP@GlcN-HA retained a spherical, membrane-enclosed morphology with an intensified bright outer layer, corroborating successful surface modification without vesicle rupture or aggregation [Figure 1B]. NTA measurements revealed that the median particle size increased from 110.2 ± 4.6 nm (TWELP) to 127.2 ± 5.6 nm (TWELP@GlcN-HA) [Figure 1C]. Following modification, the ζ-potential shifted from -15.25 ± 0.32 mV to -30.57 ± 1.21 mV, further confirming successful surface conjugation [Figure 1D]. SDS-PAGE analysis showed well-preserved TWELP protein profiles, indicating intact membrane composition after modification [Figure 1E]. Results from the DBCO-Cy5.5-based fluorescent labeling assay, particularly the competitive fluorescent labeling test [Supplementary Figure 4], verified the conjugation efficiency of DBCO-GlcN to azide-modified TWELP (TWELP-N_3_). The loading efficiencies of GlcN and HA within TWELP@GlcN-HA were determined to be 25.61% ± 4.86% and 18.25% ± 3.68%, respectively, which further confirmed the successful covalent conjugation of GlcN and efficient insertion of HA onto nanovesicles.

### Cellular uptake of TWELP@GlcN-HA

Figure 2A illustrates the cellular uptake of fluorescently labeled TWELP and engineered TWELP@GlcN-HA by macrophages. TWELP@GlcN-HA exhibited enhanced intracellular green fluorescence in LPS-activated macrophages, with higher accumulation than unmodified TWELP and non-activated macrophages, indicating that HA modification facilitates preferential uptake under inflammatory conditions. Pretreatment with excess free HA as a competitive blocking ligand significantly reduced the fluorescence intensity and cellular uptake of TWELP@GlcN-HA, as confirmed by quantitative analysis [Supplementary Figure 5]. It is well established that LPS-stimulated macrophages overexpress CD44^[30]^, and HA serves as the specific ligand for CD44 to mediate preferential nanoparticle internalization within these cells^[31]^. Collectively, these results demonstrate that TWELP@GlcN-HA uptake is largely CD44-dependent, enabling targeted accumulation in inflammation-activated macrophages and supporting its potential for precise delivery within inflammatory microenvironments^[32]^.

**Figure 2:**
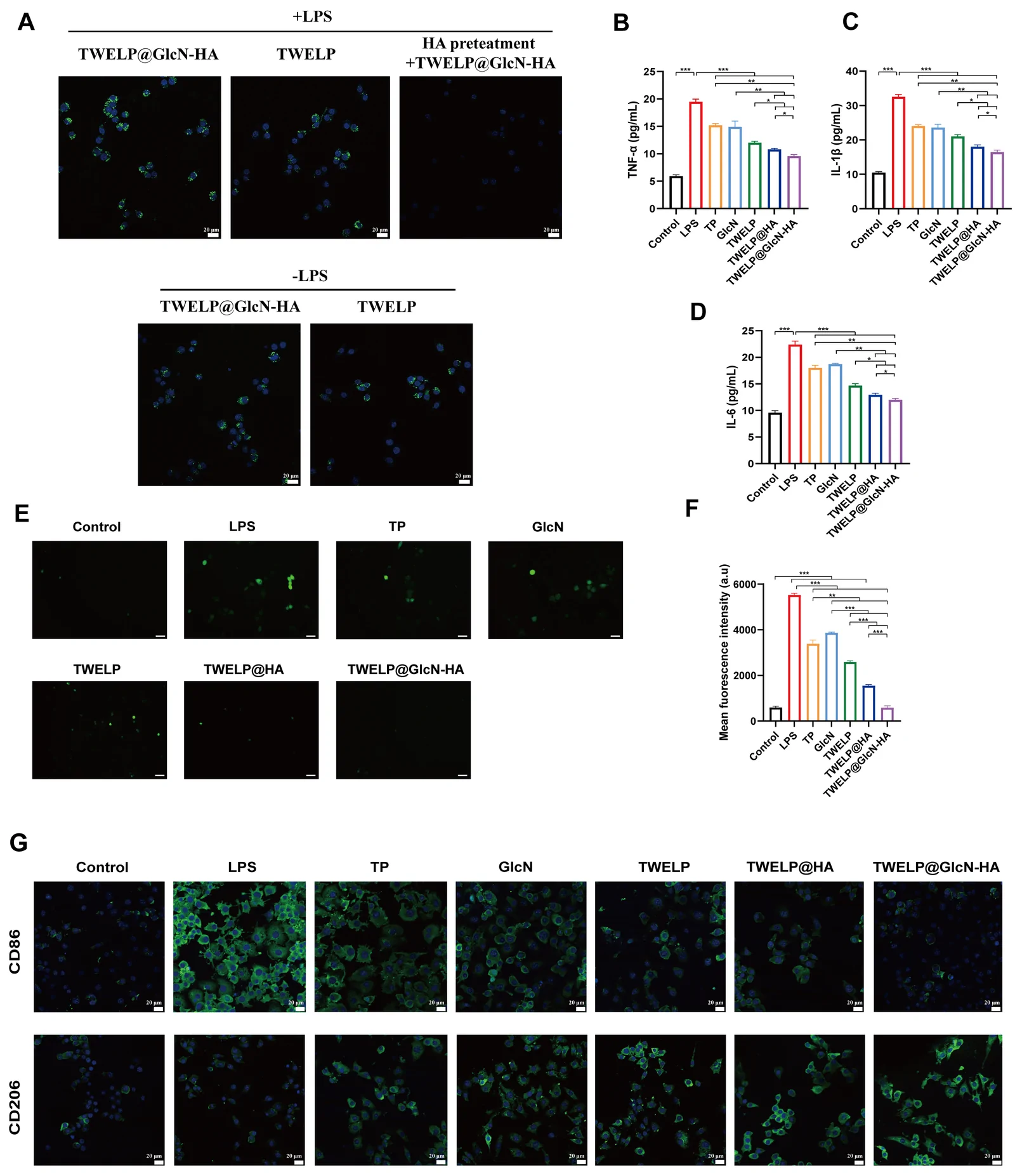
Cellular uptake, anti-inflammatory, antioxidant, and macrophage polarization effects of TWELP@GlcN-HA in RAW264.7 macrophages. (A) CLSM images of FluoExo® Green-labeled TWELP and TWELP@GlcN-HA uptake in RAW264.7 macrophages with or without LPS stimulation (scale bar: 20 μm); (B-D) Quantification of LPS-induced cytokines: TNF-α, IL-1β, and IL-6 under different treatments; (E) ROS generation (scale bar: 20 μm); (F) Quantitative analysis of intracellular ROS; (G) Immunofluorescence staining of CD86 and CD206 for M1 and M2 macrophage phenotypes under various treatments (scale bar: 20 μm). Data are presented as mean ± SD from three independent biological replicates (*n* = 3). Statistical significance was determined using one-way ANOVA. ^*^P < 0.05, ^**^P < 0.01, ^***^P < 0.001. ANOVA: Analysis of variance; CD86: cluster of differentiation 86; CLSM: confocal laser scanning microscopy; GlcN: glucosamine; HA: hyaluronic acid; IL-1β: interleukin-1β; IL-6: interleukin-6; LPS: lipopolysaccharide; ROS: reactive oxygen species; SD: standard deviation; TNF-α: tumor necrosis factor-α; TP: triptolide; TWELP: *Tripterygium wilfordii*-derived exosome-like nanoparticles; TWELP@GlcN-HA: engineered *Tripterygium wilfordii*-derived exosome-like nanoparticle system; TWELP@HA: hyaluronic acid-modified TWELP.

### *In vitro* anti-inflammatory and antioxidant activities

In RA, macrophages are highly activated within the synovial microenvironment and secrete abundant pro-inflammatory cytokines, including TNF-α, IL-1β, and IL-6, which amplify inflammation, promote FLS proliferation, and facilitate cartilage degradation and osteoclast formation^[33]^. LPS stimulation markedly increased cytokine production in RAW264.7 cells. Treatment with TP or GlcN partially reduced cytokine levels, whereas TWELP significantly suppressed TNF-α and IL-6. HA modification further enhanced anti-inflammatory efficacy, as TWELP@HA showed lower cytokine secretion than unmodified TWELP. Notably, TWELP@GlcN-HA achieved the most pronounced suppression across all three cytokines [Figure 2B-D] and concurrently reduced intracellular ROS to near-control levels [Figure 2E and F]. This indicates that the combination of TWELP’s intrinsic antioxidant and immunomodulatory components with HA-mediated targeting and GlcN delivery synergistically modulates both inflammatory cytokine secretion and oxidative stress in activated macrophages^[34,35]^. Furthermore, TWELP@GlcN-HA exerted significant inhibitory effects on HFLS-RA and MH7A cell viability [Supplementary Figure 6], as indicated by increased red fluorescence in Live/Dead staining. TP and GlcN showed minimal effect, whereas TWELP, TWELP@HA, and TWELP@GlcN-HA progressively increased cell death, consistent with suppression of pro-inflammatory cytokines and ROS. These findings highlight the potential of TWELP@GlcN-HA to attenuate inflammatory responses and protect joint tissues.

### Analysis of macrophage polarization

Macrophages undergo phenotypic remodeling within the inflammatory microenvironment in response to stimuli such as LPS, ROS, and pro-inflammatory cytokines. Their polarization toward the M1 or M2 phenotype determines whether inflammation is amplified or resolved. To assess the effects of TWELP@GlcN-HA on macrophage polarization, the expression of the M1 marker CD86 and M2 marker CD206 was examined in RAW264.7 cells [Figure 2G and Supplementary Figure 7]. CD86 signals were minimal in the control group, whereas LPS markedly increased CD86 fluorescence, indicating M1 polarization. TP and GlcN partially reduced CD86 expression, although its levels remained relatively high. TWELP, TWELP@HA, and particularly TWELP@GlcN-HA significantly suppressed CD86 expression, with TWELP@GlcN-HA producing the strongest inhibitory effect. Conversely, CD206 expression was reduced by LPS, partially restored by TP and GlcN, further enhanced by TWELP and TWELP@HA, and maximally increased by TWELP@GlcN-HA to levels approaching or exceeding baseline. These results indicate that TWELP@GlcN-HA effectively suppresses M1 polarization while promoting M2 polarization.

### Transcriptomic and metabolomic analysis of cells

Transcriptomic sequencing of LPS-activated macrophages treated with PBS, TWELP, or TWELP@GlcN-HA yielded high-quality data, as demonstrated by the fragments per kilobase of transcript per million mapped reads (FPKM) density curves [Figure 3A], and showed strong reproducibility (R > 0.9; Figure 3B). Principal component analysis revealed clear separation between the TWELP and TWELP@GlcN-HA groups, indicating substantial treatment-induced changes in gene expression [Figure 3C]. Using the predefined thresholds of |log_2_FC| > 1 and a Benjamini-Hochberg-adjusted *P* value (FDR) < 0.05, exploratory differential expression analysis identified 3,514 candidate DEGs, including 1,159 upregulated and 2,355 downregulated genes [Figure 3D and E], which were subsequently subjected to GO and KEGG enrichment analyses. The complete DEG results, including log_2_FC, raw *P* values, and Benjamini-Hochberg-adjusted *P* values (padj), are provided in Supplementary Table 1. GO enrichment analysis implicated processes related to nucleic acid metabolism, gene regulation, and transcription factor activity [Figure 3F], whereas KEGG analysis highlighted inflammation-related pathways, including the TNF, NOD-like receptor, HIF-1, and p53 signaling pathways, as well as pathways involved in the cell cycle, apoptosis, and VEGF signaling [Figure 3G]. These exploratory findings suggest that TWELP@GlcN-HA may modulate multiple pathways involved in immune homeostasis and inflammatory regulation. Metabolomic profiling identified diverse bioactive molecules, including flavonoids, fatty acids, and terpenoids [Supplementary Figures 8 and 9], which may contribute to the anti-inflammatory and immunomodulatory activities.

**Figure 3:**
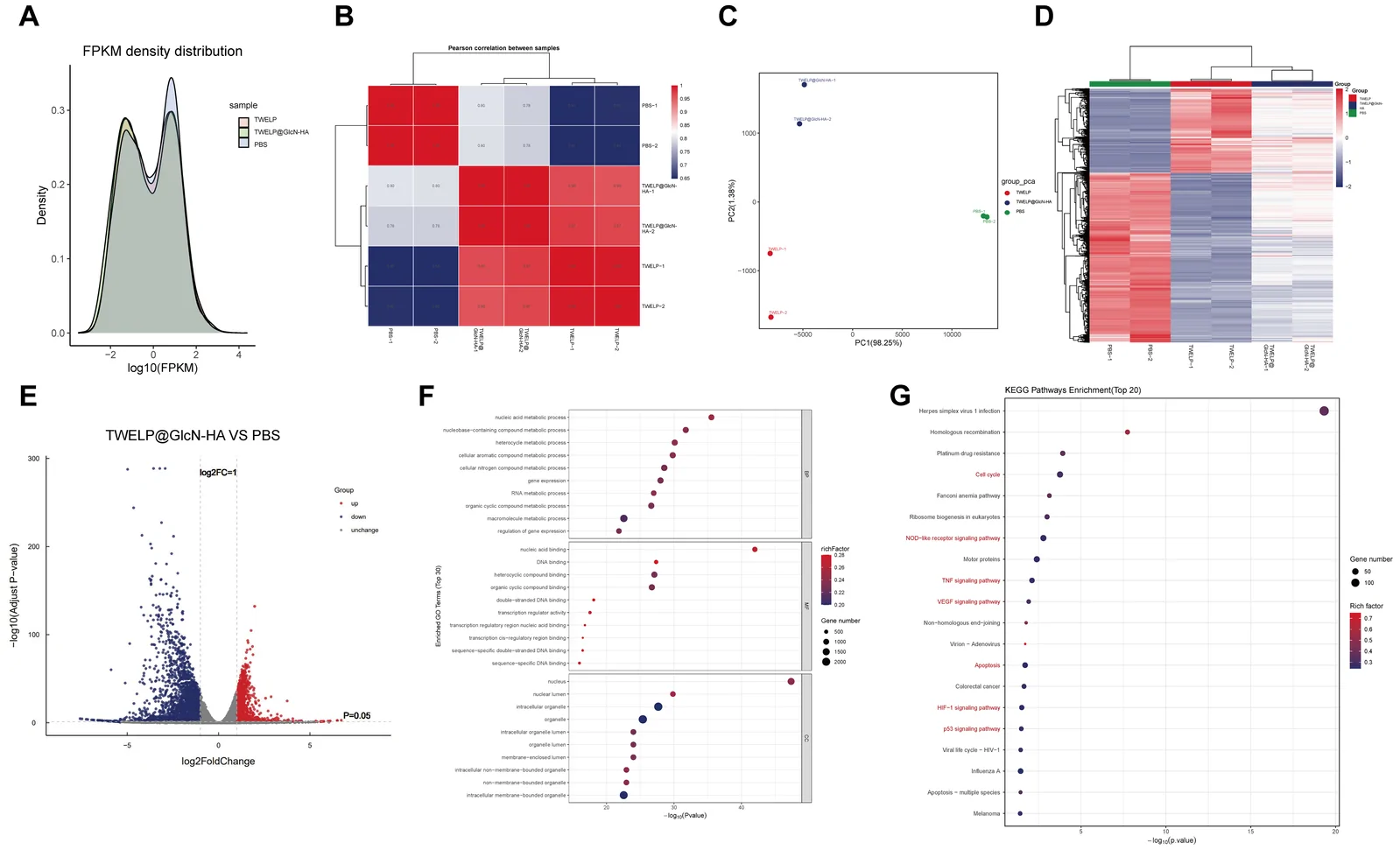
Integrated presentation of transcriptomic and metabolomic analyses. (A) FPKM density distribution of transcript expression across samples; (B) Pearson correlation heatmap showing reproducibility among replicates; (C) PCA scatter plot illustrating global expression differences; (D) Hierarchical clustering of DEGs; (E) Volcano plot showing upregulated, downregulated, and unchanged genes. Differential expression analysis was performed using DESeq2 based on raw read counts. Statistical significance was assessed using the Wald test, and *P* values were adjusted using the Benjamini-Hochberg procedure to control the FDR. Genes with |log_2_FC| > 1 and an adjusted *P* value (FDR) < 0.05 were considered DEGs; (F) GO enrichment bubble plot for BP, MF, and CC; (G) KEGG pathway enrichment bar chart of the top 20 significantly enriched pathways. GO and KEGG enrichment analyses were performed using Fisher’s exact test, with *P* values adjusted using the Benjamini-Hochberg procedure. BP: Biological process; CC: cellular component; DEGs: differentially expressed genes; FDR: false discovery rate; FPKM: fragments per kilobase of transcript per million mapped reads; GO: Gene Ontology; KEGG: Kyoto Encyclopedia of Genes and Genomes; log_2_FC: log_2_ fold change; MF: molecular function; PBS: phosphate-buffered saline; PCA: principal component analysis; TWELP: *Tripterygium wilfordii*-derived exosome-like nanoparticles; TWELP@GlcN-HA: engineered *Tripterygium wilfordii*-derived exosome-like nanoparticle system.

### *In vivo* targeting performance of TWELP@GlcN-HA

The targeting capability of TWELP@GlcN-HA was evaluated in CIA mice using DiR-labeled nanocarriers. Mice were injected intravenously with free DiR, TWELP-DiR, or TWELP@GlcN-HA-DiR (0.01 mg DiR/mouse) after joint swelling appeared. Near-infrared imaging showed weak, transient signals for free DiR, stronger and sustained signals for TWELP-DiR, and the highest, prolonged retention for TWELP@GlcN-HA, with fluorescence still visible at 72 h [Figure 4A and B]. These results indicate that GlcN and HA surface modifications enhance accumulation and retention in inflamed joints, likely via CD44-mediated recognition and inflammation-activated endocytosis.

**Figure 4:**
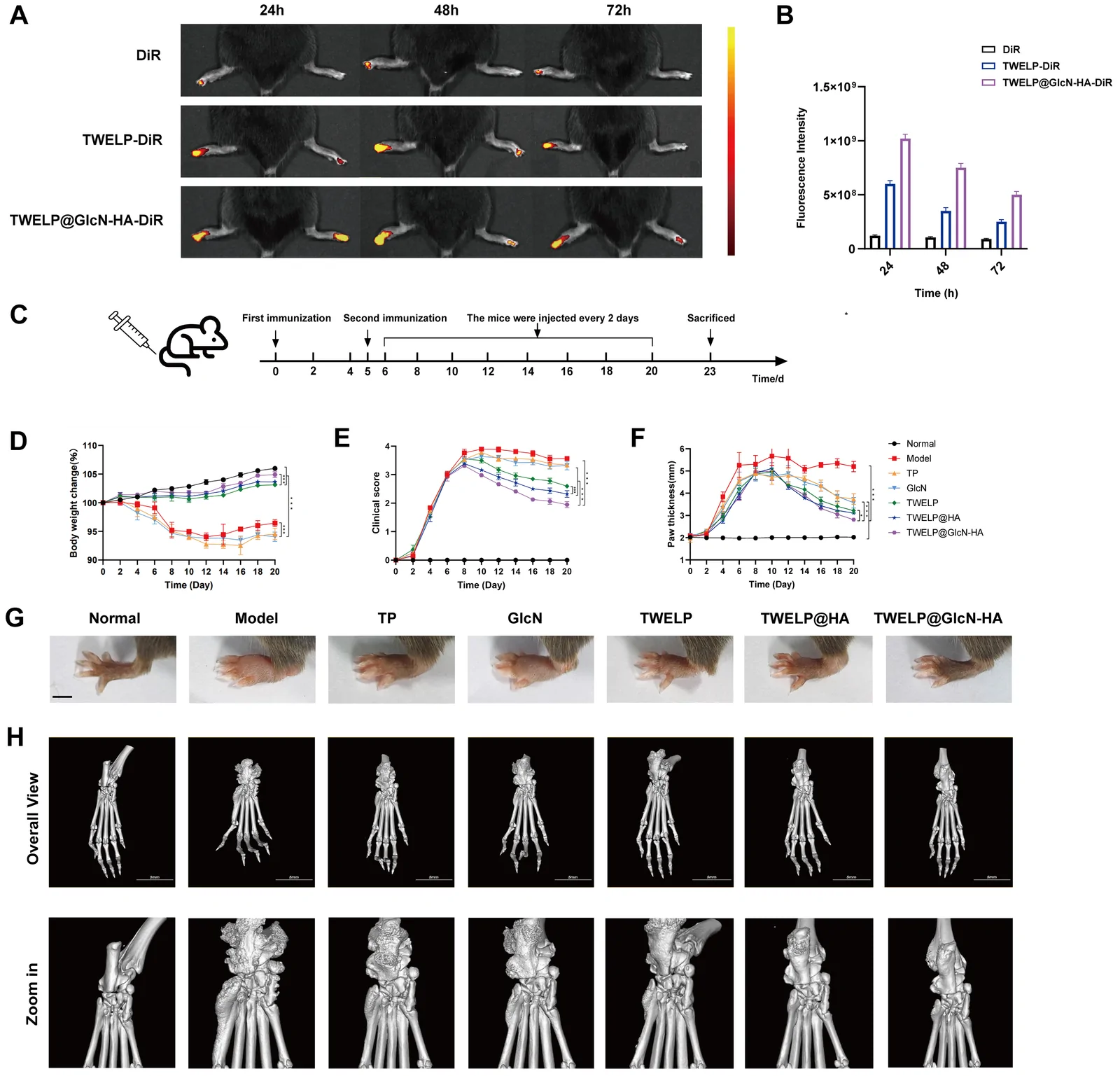
*In vivo* joint-targeting and anti-arthritic effects of TWELP@GlcN-HA in CIA mice. (A) *In vivo* fluorescence imaging at 24, 48, and 72 h after tail-vein injection of Free DiR, TWELP-DiR, or TWELP@GlcN-HA-DiR, showing stronger and sustained signals at inflamed joints for TWELP@GlcN-HA-DiR; (B) Quantitative analysis of fluorescence intensity in the inflamed paws at 24, 48, and 72 h after tail-vein injection; (C) Timeline of CIA model establishment and treatment; (D-F) Body weight, clinical arthritis scores, and hind paw thickness over time; (G) Representative hind paw images at study endpoint (scale bar: 2 mm); (H) 3D micro-CT images of the right hind limb showing bone architecture and erosion (scale bar: 5 mm). Data are presented as mean ± SD (*n* = 4). Longitudinal data were analyzed using two-way repeated-measures ANOVA, with measurements from the same animal matched across time, followed by Sidak’s multiple comparisons test. ^*^P < 0.05, ^**^P < 0.01, and ^***^P < 0.001. ANOVA: Analysis of variance; CIA: collagen-induced arthritis; DiR: 1,1’-dioctadecyl-3,3,3’,3’-tetramethylindotricarbocyanine iodide; GlcN: glucosamine; micro-CT: micro-computed tomography; SD: standard deviation; TP: triptolide; TWELP: *Tripterygium wilfordii*-derived exosome-like nanoparticles; TWELP-DiR: DiR-labeled TWELP; TWELP@GlcN-HA: engineered *Tripterygium wilfordii*-derived exosome-like nanoparticle system; TWELP@GlcN-HA-DiR: DiR-labeled TWELP@GlcN-HA; TWELP@HA: hyaluronic acid-modified TWELP.

### Therapeutic effects of TWELP@GlcN-HA in CIA mice

The therapeutic efficacy of TWELP@GlcN-HA was evaluated in CIA mice by assessing joint inflammation, bone destruction, and bone microarchitecture [Figure 4C]. Before treatment initiation on day 6, the arthritis scores were 3.01 ± 0.11, 3.04 ± 0.04, 3.01 ± 0.09, 2.99 ± 0.02, 2.95 ± 0.04, and 2.93 ± 0.15 in the Model, TP, GlcN, TWELP, TWELP@HA, and TWELP@GlcN-HA groups. No significant difference was observed among the groups at baseline, indicating comparable disease severity before treatment. At treatment initiation, the CIA mice exhibited early clinical signs of arthritis, including joint swelling and restricted mobility. TP and GlcN moderately alleviated the symptoms of arthritis, whereas TWELP produced a more pronounced therapeutic effect, likely because of the intrinsic anti-inflammatory and immunomodulatory constituents retained from *Tripterygium wilfordii*. Notably, TWELP@HA and particularly TWELP@GlcN-HA further attenuated body weight loss and reduced arthritis scores and paw thickness compared with unmodified TWELP. These findings demonstrate that surface functionalization provides additional targeting and therapeutic benefits beyond the inherent activity of TWELP [Figure 4D-G]. Macroscopic and micro-CT analyses revealed severe bone erosion in the model group, which TP, GlcN, or TWELP partially ameliorated. TWELP@GlcN-HA restored bone structure, producing dense, continuous trabeculae and minimal lesions [Figure 4H]. Micro-CT analysis showed abnormal bone remodeling in CIA mice, characterized by increased bone volume (BV) and trabecular bone surface (Tb.BS), together with decreased bone volume fraction (BV/TV), bone mineral density (BMD), and trabecular thickness (Tb.Th). TWELP@GlcN-HA partially reversed these microarchitectural abnormalities and improved trabecular bone integrity [Figure 5A-F]. Collectively, these results demonstrate that TWELP@GlcN-HA effectively suppresses arthritis progression, prevents bone erosion, and promotes structural recovery by combining the intrinsic therapeutic activity of TWELP with enhanced targeting and functional modification.

**Figure 5:**
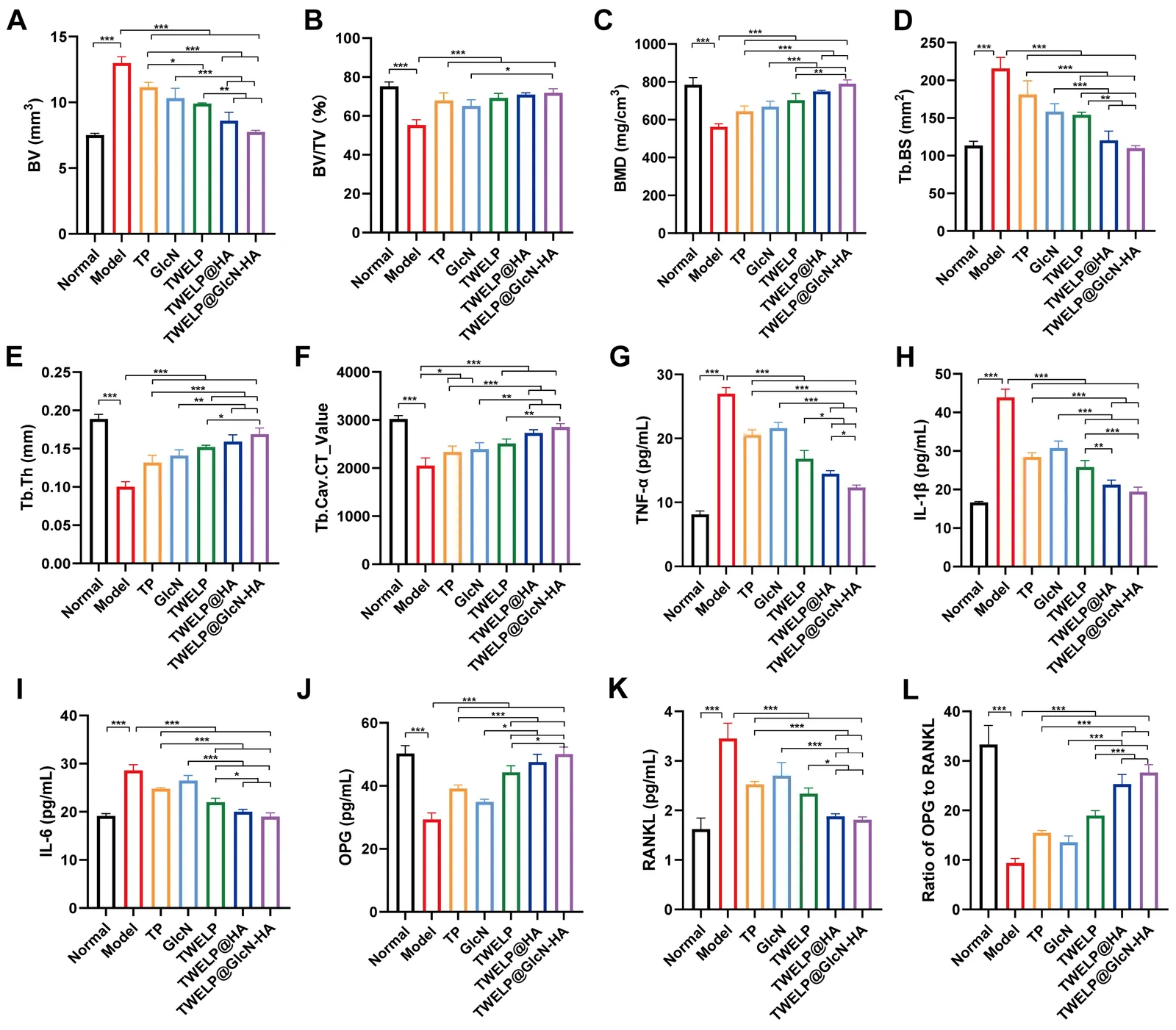
TWELP@GlcN-HA mitigates bone destruction and inflammation in CIA mice. (A-F) Quantitative micro-CT analysis of bone parameters: BV, BV/TV, BMD, Tb.BS, Tb.Th, and Tb.Cav.CT_Value; (G-I) Serum levels of pro-inflammatory cytokines TNF-α, IL-1β, and IL-6; (J-L) OPG, RANKL, and OPG/RANKL ratio reflecting bone remodeling. Data are mean ± SD; one-way ANOVA, ^*^P < 0.05, ^**^P < 0.01, ^***^P < 0.001 (*n* = 4). ANOVA: Analysis of variance; BMD: bone mineral density; BV: bone volume; BV/TV: bone volume fraction; CIA: collagen-induced arthritis; GlcN: glucosamine; IL-1β: interleukin-1β; IL-6: interleukin-6; micro-CT: micro-computed tomography; OPG: osteoprotegerin; OPG/RANKL: ratio of osteoprotegerin to receptor activator of nuclear factor-κB ligand; RANKL: receptor activator of nuclear factor-κB ligand; SD: standard deviation; Tb.BS: trabecular bone surface; Tb.Cav.CT_Value: trabecular cavity CT value; Tb.Th: trabecular thickness; TNF-α: tumor necrosis factor-α; TP: triptolide; TWELP: *Tripterygium wilfordii*-derived exosome-like nanoparticles; TWELP@GlcN-HA: engineered *Tripterygium wilfordii*-derived exosome-like nanoparticle system; TWELP@HA: hyaluronic acid-modified TWELP.

### TWELP@GlcN-HA regulates inflammatory responses and improves the bone-destructive microenvironment

Sustained macrophage activation in RA drives excessive TNF-α, IL-1β, and IL-6 production, contributing to synovial damage and joint destruction. Serum cytokines were elevated in the model group, partially reduced by TP, GlcN, or TWELP, and more strongly suppressed by TWELP@HA and TWELP@GlcN-HA, with TWELP@GlcN-HA restoring levels close to normal [Figure 5G-I]. The RANKL-RANK axis promotes osteoclast differentiation, while OPG inhibits it^[36]^. In the model, OPG decreased, RANKL increased, and the OPG/RANKL ratio declined, reflecting enhanced osteoclast activity [Figure 5J-L]. TWELP@GlcN-HA most effectively restored this balance, consistent with reports that co-delivery of TP and GlcN better modulates the OPG/RANKL axis^[37]^.

H&E staining of synovial tissue confirmed these findings [Figure 6A and B]. The model exhibited thickened synovium, inflammatory infiltration, neovascularization, and cartilage disruption, resulting in elevated histological scores. TP, GlcN, and TWELP moderately alleviated these changes, while TWELP@HA and TWELP@GlcN-HA markedly reduced synovial hyperplasia, inflammation, and partially restored cartilage, yielding the lowest HSS. Collectively, TWELP@GlcN-HA suppresses systemic inflammation, restores bone homeostasis, and promotes synovial and cartilage repair, outperforming single-drug or unmodified TWELP treatments.

**Figure 6:**
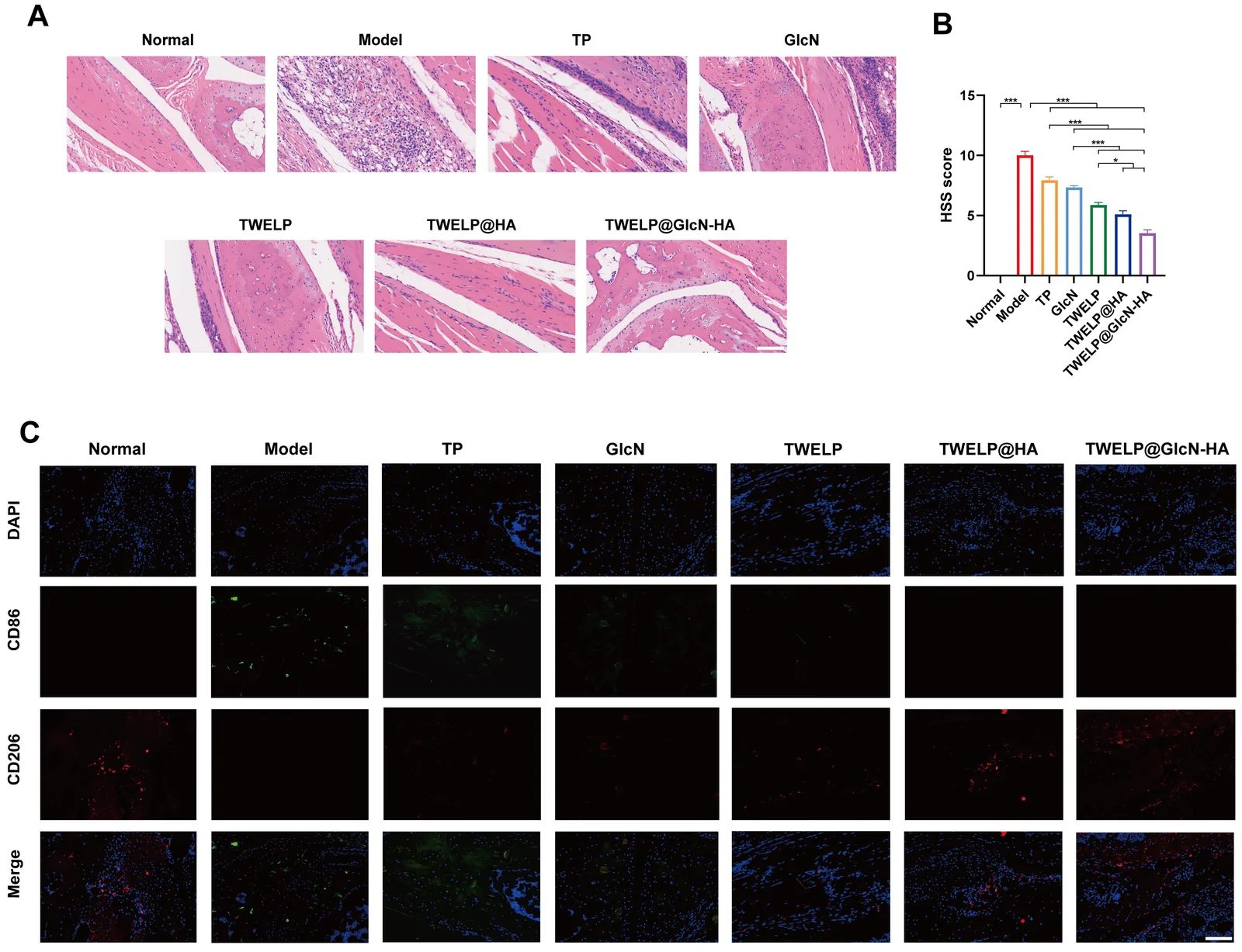
Histological evaluation and macrophage polarization in CIA mouse ankle joints after TWELP@GlcN-HA treatment. (A) Representative H&E-stained synovial images (scale bar: 100 μm); (B) HSS. Data are mean ± SD; one-way ANOVA, ^*^P < 0.05, ^**^P < 0.01, ^***^P < 0.001 (*n* = 4); (C) Immunofluorescence of M1/M2 macrophages: nuclei (DAPI, blue), M1 (CD86, green), M2 (CD206, red). Scale bar: 100 μm. ANOVA: Analysis of variance; CD86: cluster of differentiation 86; CIA: collagen-induced arthritis; DAPI: 4’,6-diamidino-2-phenylindole; GlcN: glucosamine; H&E: hematoxylin and eosin; SD: standard deviation; TP: triptolide; TWELP: *Tripterygium wilfordii*-derived exosome-like nanoparticles; TWELP@GlcN-HA: engineered *Tripterygium wilfordii*-derived exosome-like nanoparticle system; TWELP@HA: hyaluronic acid-modified TWELP; HSS: histological scoring system.

### TWELP@GlcN-HA restores the M1/M2 balance of synovial macrophages

Immunofluorescence staining [Figure 6C] showed that synovial tissues in the model group accumulated CD86^+^ M1 macrophages and had reduced CD206^+^ M2 macrophages, indicating pro-inflammatory polarization. TP, GlcN, and TWELP partially suppressed M1 and restored M2 polarization, while TWELP@HA further increased M2 macrophages. TWELP@GlcN-HA had the strongest effect, markedly reducing CD86, elevating CD206, and restoring the M1/M2 ratio toward normal. This suggests that TWELP@GlcN-HA effectively shifts macrophages toward the anti-inflammatory M2 phenotype, likely via HA-CD44-mediated targeting, enhanced GlcN delivery, and the antioxidant and immunomodulatory properties of TWELP, collectively modulating ROS and inflammatory signaling in the RA microenvironment^[38,39]^.

### Biosafety evaluation of TWELP@GlcN-HA

Free TP is known to cause hepatotoxicity, which limits its clinical use^[40]^. *In vitro*, RAW264.7 and HFLS cells treated with TWELP or TWELP@GlcN-HA maintained high viability up to 50 μg/mL, whereas TP caused dose-dependent cytotoxicity [Figure 7A], indicating good nanoparticle biocompatibility. In CIA mice, TWELP@GlcN-HA normalized hematological parameters and liver/kidney markers [alanine aminotransferase (ALT), aspartate aminotransferase (AST), blood urea nitrogen (BUN), creatinine (CREA)], which were elevated in the model and free drug groups [Figure 7B and C]. Histology showed intact heart, liver, spleen, lung, and kidney tissues with no necrosis in the TWELP@GlcN-HA group, whereas controls showed mild hepatocellular degeneration and inflammation [Figure 7D]. These results demonstrate that TWELP@GlcN-HA is safe *in vitro* and *in vivo*, supporting its potential as a nanotherapeutic for RA.

**Figure 7:**
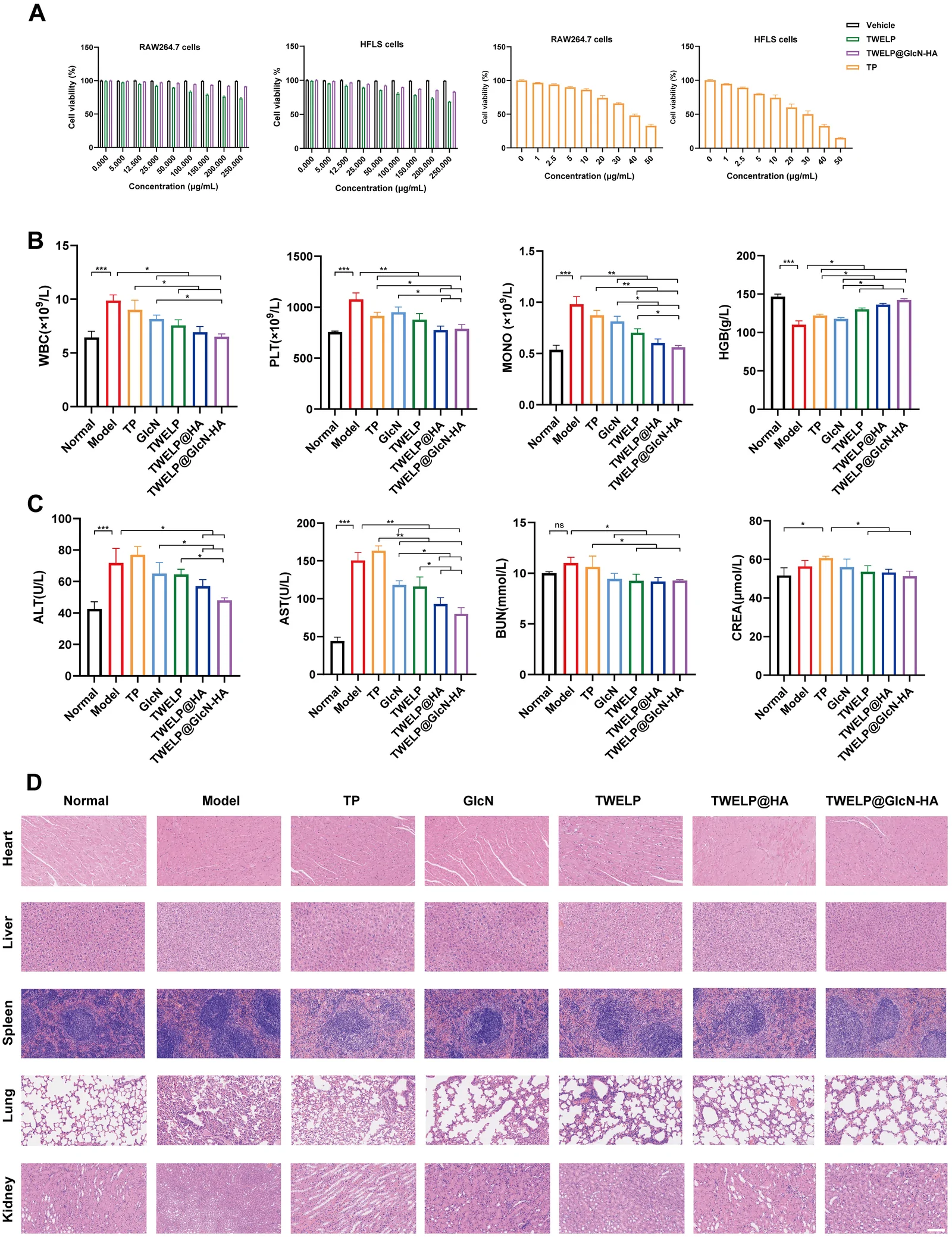
*In vitro* and *in vivo* biosafety evaluation of TWELP@GlcN-HA. (A) Cytotoxicity of Vehicle, TWELP, TWELP@GlcN-HA, and TP in RAW264.7 and HFLS cells (0-250 μg/mL for TWELP formulations and 0-50 μg/mL for TP); (B) Hematological parameters: WBC, PLT, MONO, HGB; (C) Serum biochemical markers for liver and kidney function: ALT, AST, BUN, CREA; (D) H&E staining of major organs: heart, liver, spleen, lung, kidney (scale bar: 100 μm). Data are mean ± SD (*n* = 4); one-way ANOVA, ^*^P < 0.05, ^**^P < 0.01, ^***^P < 0.001. ALT: Alanine aminotransferase; ANOVA: analysis of variance; AST: aspartate aminotransferase; BUN: blood urea nitrogen; CREA: creatinine; GlcN: glucosamine; H&E: hematoxylin and eosin; HFLS: human fibroblast-like synoviocytes; HGB: hemoglobin; MONO: monocyte; PLT: platelet; SD: standard deviation; TP: triptolide; TWELP: *Tripterygium wilfordii*-derived exosome-like nanoparticles; TWELP@GlcN-HA: engineered *Tripterygium wilfordii*-derived exosome-like nanoparticle system; TWELP@HA: hyaluronic acid-modified TWELP; WBC: white blood cell.

## DISCUSSION

In this study, we engineered *Tripterygium wilfordii*-derived exosome-like nanoparticles co-modified with GlcN and HA (TWELP@GlcN-HA) as a plant-derived nanotherapeutic platform for RA treatment. The strategy integrates three complementary functional layers: the intrinsic anti-inflammatory and antioxidant capacity of native TWELP, the cartilage-protective effects of GlcN, and the CD44-mediated active targeting conferred by HA surface modification. Together, these features enable TWELP@GlcN-HA to modulate the inflammatory microenvironment of RA through ROS regulation, macrophage reprogramming, and targeted joint accumulation.

The therapeutic rationale of TWELP@GlcN-HA is rooted in the pathological features of RA, which is characterized by excessive ROS accumulation, disruption of the macrophage M1/M2 balance, and RANKL-mediated osteoclast activation. These processes establish a self-perpetuating inflammatory microenvironment in which pro-inflammatory macrophages serve as central orchestrators^[34,41]^. Native TWELP, as a natural exosome-like carrier, is enriched in lipids and bioactive small molecules derived from *Tripterygium wilfordii* that confer intrinsic antioxidant and immunomodulatory properties. Our *in vitro* results demonstrated that TWELP@GlcN-HA efficiently scavenged ROS and suppressed TNF-α and IL-6 secretion, while promoting the polarization of macrophages from the pro-inflammatory M1 phenotype toward the anti-inflammatory M2 phenotype. These findings indicate that TWELP provides a bioactive foundation upon which GlcN and HA modifications exert synergistic enhancement.

GlcN contributes to the therapeutic effect through cartilage-protective mechanisms^[42,43]^. In the ROS-rich and mildly acidic RA microenvironment, GlcN regulates extracellular matrix metabolism, reduces MMP secretion by FLSs, and downregulates RANKL expression, thereby inhibiting osteoclast differentiation and correcting the imbalance in bone resorption. In our study, the significantly enhanced efficacy of TWELP@GlcN-HA compared with TWELP supports the contribution of GlcN to the overall anti-inflammatory activity. Notably, oral GlcN has a bioavailability of only approximately 6%, and rarely reaches joint tissues at therapeutically effective concentrations after systemic administration. The nanoparticle-mediated delivery strategy employed here circumvents this limitation by presenting GlcN on the nanoparticle surface, where it can be co-delivered with TWELP cargo directly to the inflamed joint, thereby increasing local drug concentration while reducing systemic exposure.

HA modification enhances targeted uptake in inflammatory macrophages^[44]^. CD44 is overexpressed on activated macrophages and synovial fibroblasts in inflamed joints^[45]^. HA decoration enables TWELP@GlcN-HA to recognize and accumulate preferentially in arthritic tissues, as confirmed by our *in vivo* biodistribution results showing enhanced nanoparticle retention in inflamed joints. This targeting mechanism, combined with the increased vascular permeability characteristic of inflamed tissues, prolongs local retention and improves the efficiency of drug delivery to the disease site. The biodistribution data thus provide a mechanistic explanation for the superior *in vivo* performance of TWELP@GlcN-HA over unmodified TWELP and TWELP@GlcN.

In the CIA mouse model, TWELP@GlcN-HA significantly alleviated synovial inflammation, cartilage damage, and bone erosion, while improving joint morphology and function. These therapeutic effects were accompanied by a shift in the macrophage polarization balance toward the M2 phenotype and reduced levels of pro-inflammatory cytokines in joint tissues, consistent with the *in vitro* immunomodulatory activity. Importantly, no systemic toxicity or organ damage was observed, supporting the biocompatibility of this plant-derived nanoplatform. Compared with conventional synthetic nanocarriers such as poly(lactic-co-glycolic acid) (PLGA) nanoparticles and liposomes, TWELP offer several inherent advantages, including a natural lipid bilayer structure, high biocompatibility, low immunogenicity, and intrinsic antioxidant and immunoregulatory activities, embodying the concept of a "carrier-as-therapeutic" platform^[46-48]^.

Several limitations of this study should be acknowledged. First, although the therapeutic effects of TWELP@GlcN-HA were consistently supported by clinical scoring, micro-CT analysis, cytokine quantification, histological evaluation, and macrophage polarization assessment, the current CIA animal experiments represent preliminary proof-of-concept studies with a relatively limited sample size and an early therapeutic intervention paradigm. Future studies with prospectively calculated sample sizes, larger animal cohorts, prolonged modeling periods, and treatment initiated after fully established arthritis are warranted to further validate therapeutic efficacy and long-term safety. Second, the mechanistic investigation focused primarily on macrophage polarization and inflammatory regulation, whereas RA involves complex cellular crosstalk among synovial fibroblasts, dendritic cells, T lymphocytes, neutrophils, and osteoclast-lineage cells. Extracellular vesicles (EVs) may also participate in such intercellular communication, and recent evidence indicates that extracellular microvesicles from patients with RA can promote dendritic cell activation and amplify inflammatory signaling^[49]^. In addition, interactions among autophagy, extracellular vesicle biogenesis, lipid-raft organization, and post-translational protein modifications have been implicated in RA pathogenesis and immune dysregulation^[50,51]^. Therefore, comprehensive immune profiling and mechanistic studies involving these cellular and molecular pathways are needed to more fully elucidate the therapeutic mechanisms of TWELP@GlcN-HA. Third, the transcriptomic findings provide an exploratory molecular perspective on the therapeutic effects of TWELP@GlcN-HA. Future studies incorporating expanded biological replication and complementary molecular validation will help further substantiate the identified differentially expressed genes and associated signaling pathways. Finally, although the current physicochemical and fluorescence-based analyses support successful GlcN/HA surface functionalization and enhanced targeting, additional orthogonal characterization and quantitative validation of surface modification efficiency, labeling specificity, and HA/CD44-dependent targeting would further strengthen the mechanistic evidence. Addressing these limitations will provide a more rigorous foundation for the preclinical development and future translation of TWELP@GlcN-HA.

In summary, TWELP@GlcN-HA represents a plant-derived, multifunctional nanotherapeutic platform that modulates the RA inflammatory microenvironment through ROS scavenging, M2 macrophage polarization, and HA-mediated targeted joint accumulation. This combinatorial strategy - integrating intrinsic TWELP bioactivity with GlcN-mediated cartilage-protective effects by targeted delivery - provides a promising proof-of-concept approach for RA treatment and highlights the potential of naturally derived nanocarriers for managing autoimmune diseases.

## DECLARATIONS

### Availability of data and materials

The datasets generated and/or analyzed during the current study are available from the corresponding author upon reasonable request.

### AI and AI-assisted tools statement

During the preparation of this manuscript, the AI tool ChatGPT (GPT-5.4, released 2026-03-05) was used solely for language editing. The tool did not influence the study design, data collection, analysis, interpretation, or the scientific content of the work. All authors take full responsibility for the accuracy, integrity, and final content of the manuscript.

### Copyright

© The Author(s) 2026.
