# Supplementary material for: Engineering Tripterygium wilfordii-derived exosome-like nanoparticles for targeted therapy in rheumatoid arthritis
Source: Extracell Vesicles Circ Nucl Acids. 2026 Sep 21;7(3):1534–53. doi: 10.20517/evcna.2026.66 (PMC13673461; doi:10.20517/evcna.2026.66)
Supplement: Supplementary file 1 [file evcna-7-3-1534-SupplementaryMaterials.zip › evcna7066-SupplementaryMaterials/evcna7066-SupplementaryMaterials.pdf]

Supplementary Materials

Engineering *Tripterygium wilfordii*-derived exosome-like nanoparticles for targeted therapy in rheumatoid arthritis

Yunyi Yang[1,#], Zhi Gong[2,#], Gongming Shi[3], Yong Yang[3], Yun He[2], Hongjie Yang[1], Dajian Yang[4], Xiaohong Yang[5]

[1]Yueyang Hospital of Integrated Traditional Chinese and Western Medicine, Shanghai University of Traditional Chinese Medicine, Shanghai 200437, China.

[2]Translational Innovation Center, Shenzhen Bay Laboratory, Shenzhen 518132, Guangdong, China.

[3]Chongqing Institute of Medicinal Plant Cultivation, Chongqing 408435, China.

[4]Chongqing Academy of Chinese Materia Medica, Chongqing 400065, China.

[5]Chongqing Institute of Green and Intelligent Technology, Chinese Academy of Sciences, Chongqing 400714, China.

[#]These authors contributed equally to this work.

Correspondence to: Dajian Yang, Chongqing Academy of Chinese Materia Medica, Chongqing 400065, China. E-mail: yangdajian@cqacmm.com; Xiaohong Yang, Chongqing Institute of Green and Intelligent Technology, Chinese Academy of Sciences, Chongqing 400714, China. E-mail: yangxiaohong@cigit.ac.cn

Table of contents

Section 1 Experimental materials and methods ………………………………………………...3

Section 2 Supporting figures…………..…………………………………………………………...7

Section 3 ^{1}H and ^{13}C NMR Spectrum of key Compounds……………………………………...12

1. Experimental materials and methods

1.1. Chemistry

Synthesis of DBCO-GlcN

DBCO-GlcN was synthesized through a multi-step sequence (Fig. S1) with intermediates labeled as compounds 1–9. The synthesis began with O-alkylation of 2,4-dihydroxybenzaldehyde (1) using tert-butyl bromoacetate, followed by acid-mediated deprotection to yield a carboxylic acid intermediate (3). HATU/DIPEA-mediated amidation between compound 4 and Boc-protected amine 5 produced compound 6, which was deprotected with trifluoroacetic acid (TFA) to give compound 7. Conjugation of acid 3 with amine 7 using HATU afforded intermediate 8, and a final Schiff base reaction with D-glucosamine hydrochloride yielded DBCO-GlcN (9) as a yellow syrupy solid, purified by silica gel column chromatography. The structure was confirmed by ^{1}H NMR, ^{13}C NMR, and high-resolution mass spectrometry (HRMS) (Fig. S2).

Synthesis of DSPE-PEG$_{2000}$-HA

DSPE-PEG$_{2000}$-HA was synthesized following a modified literature procedure[1, 2] (Fig. S1). Hyaluronic acid (100 mg, 3.3 μmol), NHS (3.8 mg, 33.3 μmol), and EDCI (9.6 mg, 50 μmol) were dissolved in distilled water and stirred at room temperature for 6 h. DSPE-PEG$_{2000}$-NH$_2$ (18 mg, 16.7 μmol) and a catalytic amount of DMAP in DMF (2 mL) were then added, and the reaction proceeded at room temperature for 2 days. The product was purified by dialysis (MWCO 10 kDa) against distilled water for 3 days, followed by lyophilization to yield DSPE-PEG$_{2000}$-HA as a white solid (63 mg, 53%). Successful conjugation was confirmed by ^{1}H NMR (Fig. S3).

Preparation of Cy5.5-labeled TWELP and GlcN-TWELP

To confirm the successful conjugation of TWELPs and azide ligands, TWELP-N$_3$ (1 mg/mL) was co-incubated with increased concentrations of fluorescent DBCO-Cy5.5 (0-1000 μM) at 4 ◦C for 2 h on a rotating mixer. The unconjugated ligand was removed by 100-kDa ultrafiltration tubes. The Cy5.5 conjugation efficiency was measured by a SpectraMax® i3x microplate reader (SpectraMax i3x, Molecular Device, UK) and their fluorescence intensities were normalizing TWELPs protein concentration to 1 mg/mL.

To further confirm the successful conjugation of GlcN onto the vesicle surface via click chemistry, a competitive fluorescence labeling assay was performed. Briefly, TWELP-N$_3$ was first incubated with an excess of DBCO-GlcN (1 mM) to ensure saturation of the surface azide groups. Subsequently, the reaction mixture was treated with a sufficient amount of DBCO-Cy5.5 (1 mM) to label any remaining unreacted azide moieties. Finally, the unconjugated ligands were removed by 100-kDa ultrafiltration

tubes and the fluorescence intensities of TWELP were determined by a microplate reader (Fig. S4).

Preparation of TWELP@HA

To prepare the HA-modified control formulation (TWELP@HA), equal volumes of TWELP dispersion (1 mg/mL, based on total protein) and DSPE-PEG$_{2000}$-HA solution (1 mg/mL) were mixed and incubated at room temperature for 1 h to allow the hydrophobic insertion of the DSPE moiety into the TWELP membrane. The resulting TWELP@HA was purified by ultrafiltration (MWCO 100 kDa) to remove unbound DSPE-PEG$_{2000}$-HA and was subsequently resuspended in PBS.

*Synthesis of Compound **2***

A mixture of 2,4-dihydroxybenzaldehyde (**1**) (5.0 g, 36.2 mmol, 1.0 equiv.) and *tert*-butyl bromoacetate (7.1 g, 36.2 mmol, 1.0 equiv.) in DMF (50 mL) was treated with potassium carbonate (6.0 g, 43.4 mmol, 1.5 equiv.). The reaction mixture was heated to 60 °C and stirred for 10 hours. After completion, the mixture was acidified to pH ~3 by the addition of 1 N HCl. The resulting mixture was extracted with ethyl acetate (3 × 50 mL). The combined organic extracts were concentrated under reduced pressure and the residue was purified by silica gel column chromatography (eluent: petroleum ether/ethyl acetate = 10:1 to 5:1) to afford compound **2** as a pale-yellow solid (5.1 g, 56%). **^{1}H NMR** (600 MHz, Chloroform-*d*) δ 11.35 (s, 1H), 9.63 (s, 1H), 7.37 (d, J = 8.7 Hz, 1H), 6.48 (dd, J = 8.7, 2.4 Hz, 1H), 6.29 (d, J = 2.4 Hz, 1H), 4.49 (s, 2H), 1.41 (s, 9H). **^{13}C NMR** (150 MHz, Chloroform-*d*) δ 194.47, 166.76, 164.78, 164.09, 135.34, 115.59, 108.24, 101.38, 82.76, 65.34, 27.87.

*Synthesis of Compound **3***

Compound **2** (1.0 g, 4.0 mmol) was dissolved in DCM (10 mL), followed by the addition of TFA (10 mL). The reaction mixture was stirred at room temperature for 3 hours. Upon completion, the mixture was concentrated directly under vacuum without further purification to give compound **3** as an off-white solid. **^{1}H NMR** (400 MHz, DMSO-d_6) δ 12.52–10.45 (m, 2H), 10.02 (d, J = 1.9 Hz, 1H), 7.62 (d, J = 8.7 Hz, 1H), 6.55 (dd, J = 8.7, 2.4 Hz, 1H), 6.44 (d, J = 2.4 Hz, 1H), 4.76 (s, 2H). **^{13}C NMR** (100 MHz, DMSO-d_6) δ 190.95, 169.60, 164.36, 162.89, 132.06, 116.67, 116.65, 107.63, 101.55, 64.67.

*Synthesis of Compound **6***

A solution of compound **4** (200 mg, 655.02 µmol, 1.0 equiv.), HATU (300 mg, 786.03 µmol, 1.5 equiv.), and DIPEA (169 mg, 1.31 mmol, 2.0 equiv.) in DCM (10 mL) was stirred for 20 minutes. Compound **5** (179 mg, 720.52 µmol, 1.1 equiv.) was then added, and the resulting mixture was stirred at room temperature for 3 hours. After completion, the reaction mixture was washed with 1 N HCl (10 mL). The organic phase was concentrated under reduced pressure and the residue was purified by silica gel column chromatography (eluent: DCM/methanol = 20:1 to 10:1) to afford compound **6** as a syrupy solid

(323 mg, 92%). **^{1}H NMR** (600 MHz, Chloroform-*d*) *δ* 7.65 (dd, *J* = 7.6, 1.3 Hz, 1H), 7.49 (d, *J* = 6.3 Hz, 1H), 7.40–7.34 (m, 3H), 7.32 (td, *J* = 7.5, 1.5 Hz, 1H), 7.27 (td, *J* = 7.5, 1.4 Hz, 1H), 7.22 (dd, *J* = 7.6, 1.5 Hz, 1H), 6.16 (s, 1H), 5.13 (d, *J* = 13.9 Hz, 1H), 5.08 (s, 1H), 3.64 (d, *J* = 13.9 Hz, 1H), 3.59–3.52 (m, 4H), 3.50 (t, *J* = 5.2 Hz, 2H), 3.48–3.38 (m, 2H), 3.35–3.29 (m, 2H), 3.29–3.23 (m, 2H), 2.79 (dt, *J* = 17.1, 6.1 Hz, 1H), 2.48–2.36 (m, 1H), 2.16 (dt, *J* = 15.1, 6.2 Hz, 1H), 1.93 (dt, *J* = 16.9, 6.1 Hz, 1H), 1.40 (s, 9H). **^{13}C NMR** (150 MHz, Chloroform-*d*) *δ* 172.36, 172.20, 156.08, 151.42, 148.13, 132.28, 129.42, 128.70, 128.19, 127.76, 127.10, 125.54, 123.24, 122.51, 114.75, 107.93, 79.27, 70.24, 69.78, 55.58, 40.38, 39.21, 31.26, 30.23, 28.47.

Synthesis of Compound 7

Compound **6** (323 mg, 603.02 μmol) was dissolved in DCM (5 mL), and TFA (1 mL) was added. The reaction was stirred at room temperature for 2 hours. The mixture was then concentrated directly under reduced pressure to yield compound **7**, which was used directly in the next step without further purification due to its instability. **^{1}H NMR** (400 MHz, Chloroform-*d*) *δ* 7.64–7.53 (m, 2H), 7.42–7.33 (m, 4H), 7.31–7.27 (m, 2H), 5.09 (d, *J* = 13.9 Hz, 1H), 4.88 (s, 1H), 3.67–3.45 (m, 10H), 3.32–3.19 (m, 2H), 2.96–2.89 (m, 2H), 2.85 (dt, *J* = 17.3, 5.5 Hz, 1H), 2.51 (dt, *J* = 15.1, 5.2 Hz, 1H), 2.19 (dt, *J* = 15.8, 5.7 Hz, 1H), 1.87 (dt, *J* = 17.0, 5.5 Hz, 1H). **^{13}C NMR** (100 MHz, Chloroform-*d*) *δ* 173.24, 172.47, 151.21, 148.19, 131.88, 129.52, 128.85, 128.46, 127.94, 127.90, 127.18, 125.88, 123.42, 122.56, 114.62, 107.92, 70.38, 69.96, 69.75, 68.97, 55.85, 40.63, 39.56, 30.69, 29.80.

*Synthesis of Compound **8***

A solution of compound **3** (100 mg, 505.14 μmol, 1.1 equiv.), HATU (262 mg, 688.83 μmol, 1.5 equiv.), and DIPEA (178 mg, 1.38 mmol, 3.0 equiv.) in DCM (10 mL) was stirred for 20 minutes. Compound **7** (200 mg, 459.22 μmol, 1.0 equiv.) was then added, and the resulting mixture was stirred at room temperature for 4 hours. After completion, the reaction mixture was washed with 1 N HCl (10 mL). The organic phase was concentrated under reduced pressure and the residue was purified by silica gel column chromatography (eluent: DCM/methanol = 20:1 to 10:1) to afford compound **8** as a syrupy solid (152 mg, 54%). **^{1}H NMR** (600 MHz, Chloroform-*d*) *δ* 11.40 (s, 1H), 9.75 (s, 1H), 7.65 (d, *J* = 7.5 Hz, 1H), 7.52–7.46 (m, 2H), 7.41–7.36 (m, 3H), 7.33 (t, *J* = 7.5 Hz, 1H), 7.29 (t, *J* = 7.5 Hz, 1H), 7.24 (d, *J* = 7.4 Hz, 1H), 7.05 (t, *J* = 5.7 Hz, 1H), 6.56 (dd, *J* = 8.7, 2.3 Hz, 1H), 6.44 (d, *J* = 2.3 Hz, 1H), 6.29 (s, 1H), 5.13 (d, *J* = 13.9 Hz, 1H), 4.52 (s, 2H), 3.66 (d, *J* = 13.8 Hz, 1H), 3.60–3.56 (m, 4H), 3.56–3.51 (m, 4H), 3.47 (dt, *J* = 10.3, 5.2 Hz, 1H), 3.42 (dt, *J* = 10.0, 5.3 Hz, 1H), 3.33 (t, *J* = 5.5 Hz, 2H), 2.80 (dt, *J* = 16.8, 5.8 Hz, 1H), 2.43 (dt, *J* = 14.7, 5.7 Hz, 1H), 2.19 (dt, *J* = 15.2, 6.1 Hz, 1H), 1.95 (dt, *J* = 17.1, 6.1 Hz, 1H). **^{13}C NMR** (150 MHz, Chloroform-*d*) *δ* 194.68, 172.49, 172.35, 167.17, 164.31, 164.07, 151.32, 148.09, 135.73, 132.27, 129.40, 128.77, 128.33, 128.21, 127.83, 127.18, 125.59, 123.26, 122.53,

116.17, 114.75, 108.10, 107.94, 102.21, 70.35, 70.26, 69.78, 69.70, 67.35, 55.66, 39.29, 39.00, 31.25, 30.27.

*Synthesis of Compound DBCO-GlcN (**9**)*

A mixture of compound **8** (100 mg, 162.95 μmol, 1.0 equiv.) and glucosamine hydrochloride (53 mg, 244.43 μmol, 1.5 equiv.) in ethanol (5 mL) was treated with DIPEA (42 mg, 325.91 μmol, 2.0 equiv.). The reaction mixture was heated to 80 °C and stirred for 5 hours. After completion, the mixture was concentrated under reduced pressure. The crude product was purified by silica gel column chromatography to yield DBCO-GlcN (**9**) as a yellow syrupy solid (91 mg, 72%). **^{1}H NMR** (600 MHz, Methanol-d_4) δ 8.30 (s, 0.3H), 8.15 (s, 0.7H), 7.63 (d, J = 7.5 Hz, 1H), 7.58 (d, J = 6.7 Hz, 1H), 7.47–7.41 (m, 3H), 7.34 (t, J = 7.4 Hz, 1H), 7.31 (t, J = 7.4 Hz, 1H), 7.26 (d, J = 8.7 Hz, 0.3H), 7.23 (d, J = 7.4 Hz, 1H), 7.16 (d, J = 8.8 Hz, 0.7H), 6.46 (dd, J = 8.6, 2.4 Hz, 0.3H), 6.37 (d, J = 2.4 Hz, 0.3H), 6.28 (dd, J = 8.9, 2.4 Hz, 0.7H), 6.14 (d, J = 2.4 Hz, 0.7H), 5.27 (d, J = 3.5 Hz, 0.7H), 5.11 (d, J = 14.1 Hz, 1H), 4.82 (d, J = 7.9 Hz, 0.3H), 4.51 (s, 0.6H), 4.51 (s, 1.4H), 4.49 (s, 1H), 3.93–3.88 (m, 1H), 3.84–3.81 (m, 1H), 3.78–3.71 (m, 1H), 3.68 (d, J = 14.2 Hz, 1H), 3.66–3.59 (m, 1H), 3.57–3.54 (m, 4H), 3.53–3.51 (m, 2H), 3.44 (t, J = 5.4 Hz, 2H), 3.43–3.39 (m, 4H), 3.22 (t, J = 5.5 Hz, 2H), 2.67 (dt, J = 15.8, 7.5 Hz, 1H), 2.33 (dt, J = 15.1, 7.5 Hz, 1H), 2.16 (dt, J = 15.3, 7.0 Hz, 1H), 1.96 (dt, J = 15.5, 6.8 Hz, 1H). **^{13}C NMR** (150 MHz, Methanol-d_4) δ 178.03, 174.59, 174.00, 170.65, 170.45, 168.12, 167.81, 166.50, 166.03, 163.64, 152.66, 149.46, 136.61, 135.05, 133.45, 130.84, 130.58, 129.98, 129.63, 129.17, 128.86, 128.09, 126.45, 124.37, 123.69, 115.57, 114.47, 112.61, 108.78, 107.49, 104.62, 103.52, 96.86, 92.73, 78.13, 76.31, 75.75, 73.33, 73.06, 71.80, 71.68, 71.28, 71.26, 70.44, 70.42, 68.13, 68.02, 67.80, 62.84, 62.58, 56.69, 40.31, 40.01, 31.88, 31.35

2. Supporting figures

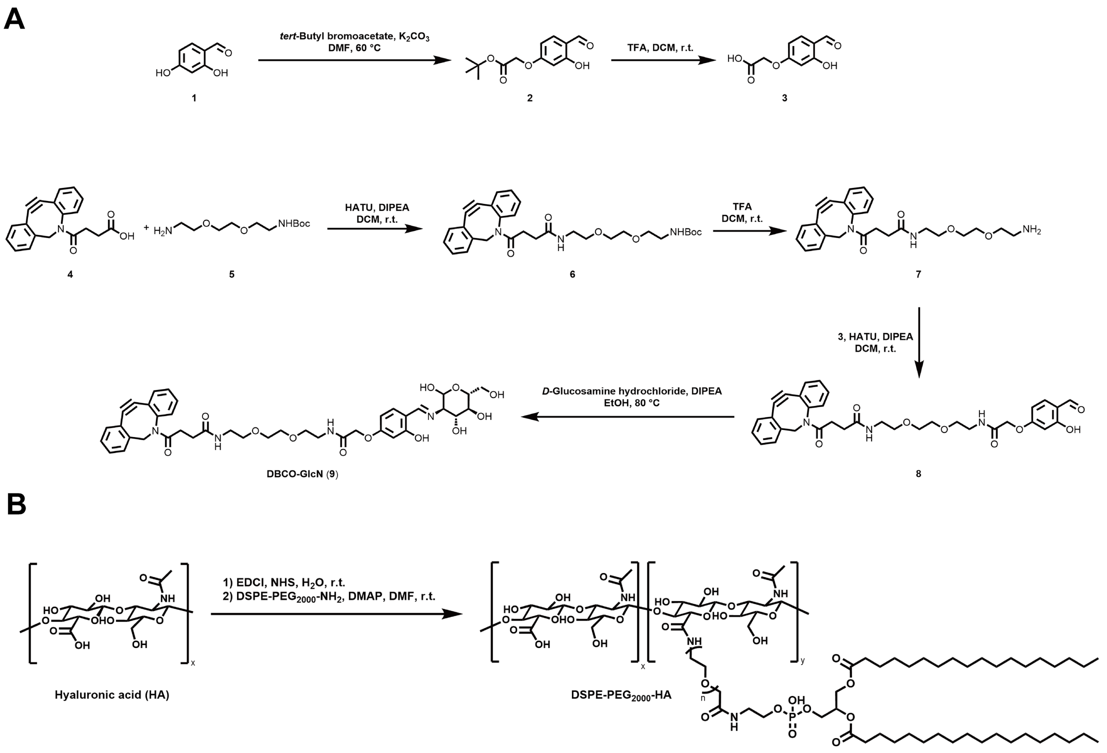

Supplementary Figure 1. Synthetic routes for DBCO-GlcN and DSPE-PEG$_{2000}$-HA. (A) Multistep synthesis of DBCO-functionalized glucosamine (DBCO-GlcN, compound 9). (B) Synthesis of DSPE-PEG$_{2000}$-conjugated hyaluronic acid (DSPE-PEG$_{2000}$-HA) through EDCI/NHS-mediated coupling of hyaluronic acid (HA) with DSPE-PEG$_{2000}$-NH$_2$.

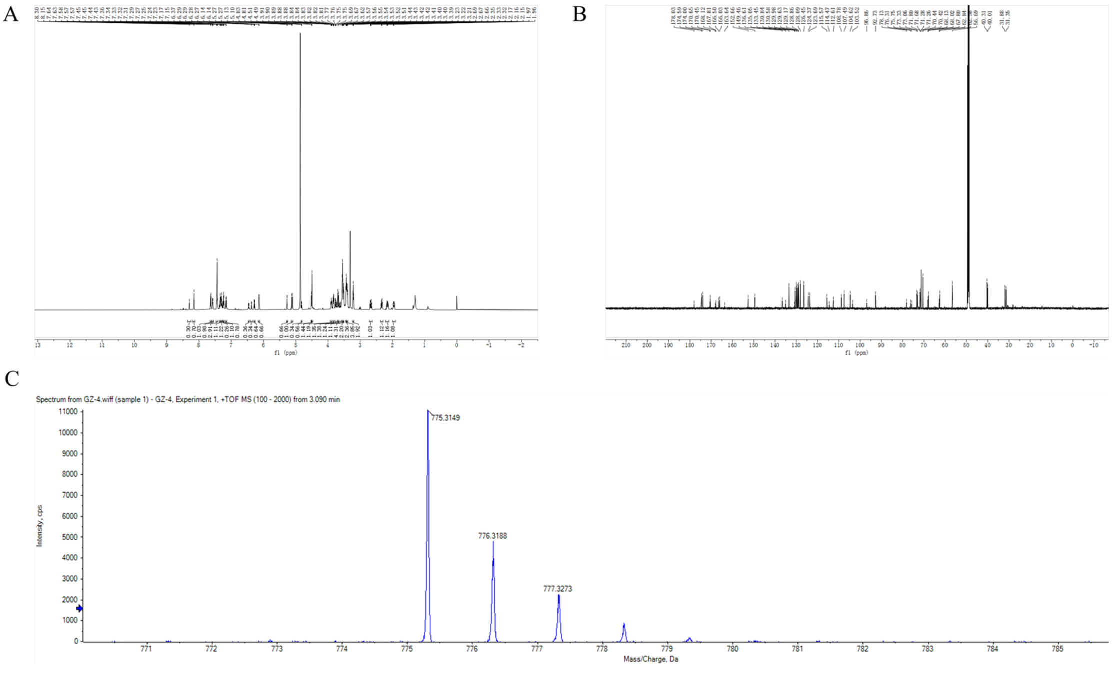

Supplementary Figure 2. Characterization of DBCO-GlcN. (A) ^{1}H NMR spectrum. (B) ^{13}C NMR spectrum. (C) High-resolution mass spectrometry spectrum.

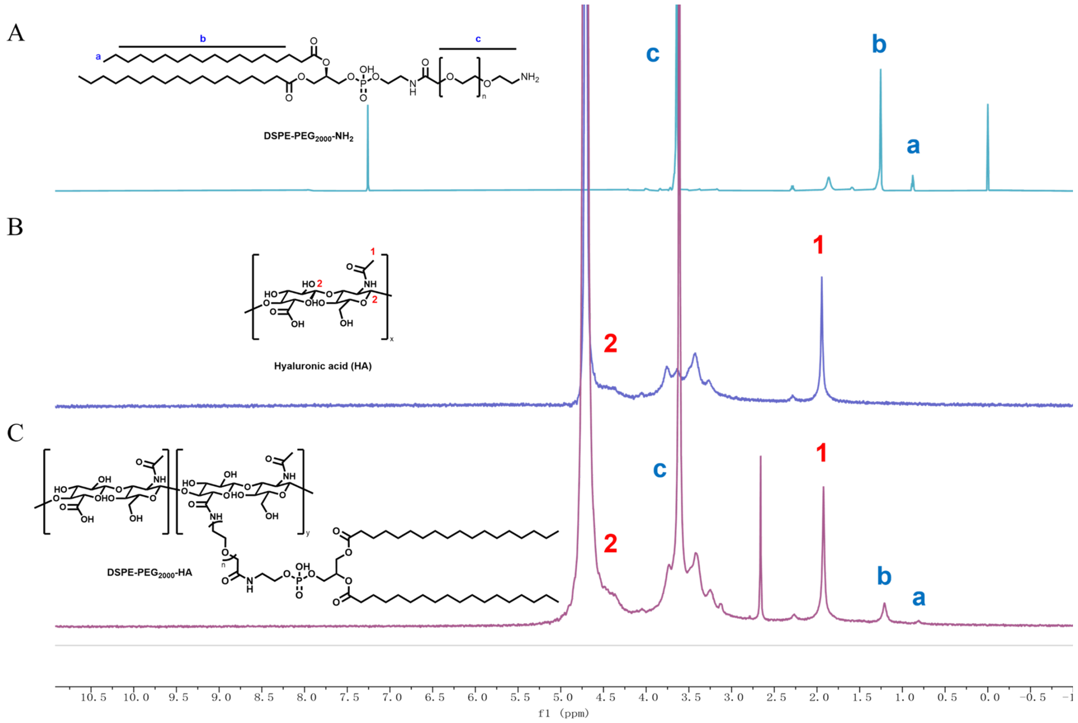

Supplementary Figure 3. Characterization of DSPE-PEG$_{2000}$-HA. (A) ^{1}H NMR spectrum of DSPE-PEG$_{2000}$-NH$_2$. (B) ^{1}H NMR spectrum of HA. (C) ^{1}H NMR spectrum of DSPE-PEG$_{2000}$-HA.

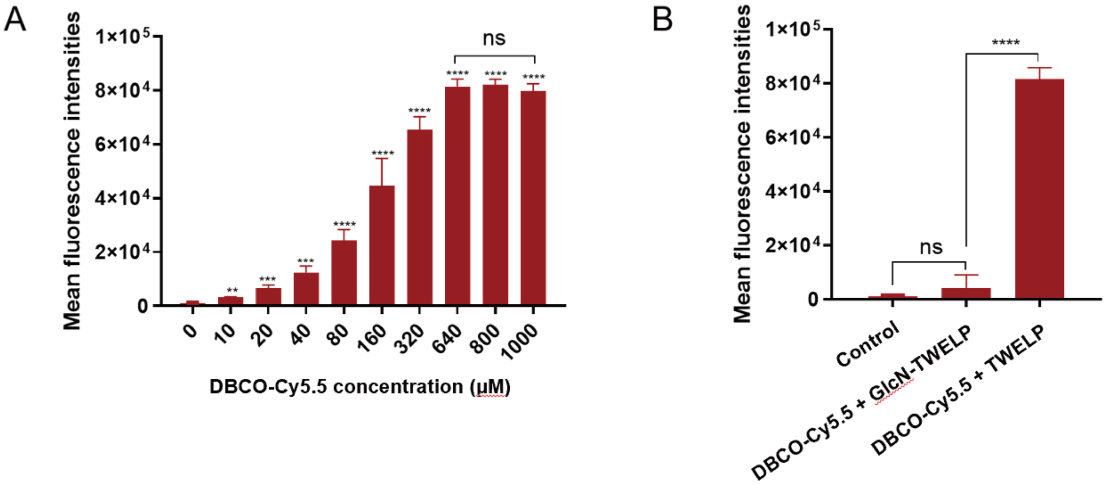

Supplementary Figure 4. Mean fluorescence intensity of Cy5.5 following the conjugation of DBCO-Cy5.5 or DBCO-GlcN with azide-functionalized TWELP (TWELP-N$_3$). (A) Cy5.5 fluorescence intensity of TWELP-N$_3$ after conjugation with increasing concentrations of DBCO-Cy5.5. (B) Comparison of Cy5.5 fluorescence intensity among the control, DBCO-Cy5.5-labeled GlcN-TWELP, and DBCO-Cy5.5-labeled TWELP groups.

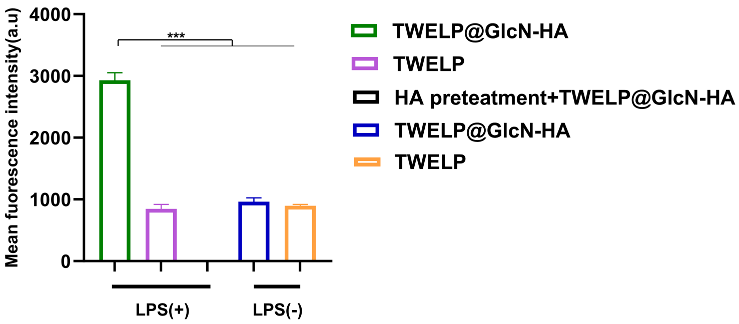

Supplementary Figure 5. Quantitative analysis of mean fluorescence intensity (MFI) of FluoExo Green-labeled TWELP and TWELP@GlcN-HA in RAW264.7 macrophages. Pretreatment with free HA reduced the uptake of TWELP@GlcN-HA by LPS-activated macrophages. Data are presented as mean ± SD from three independent biological replicates (n = 3). $^{***}P < 0.001$.

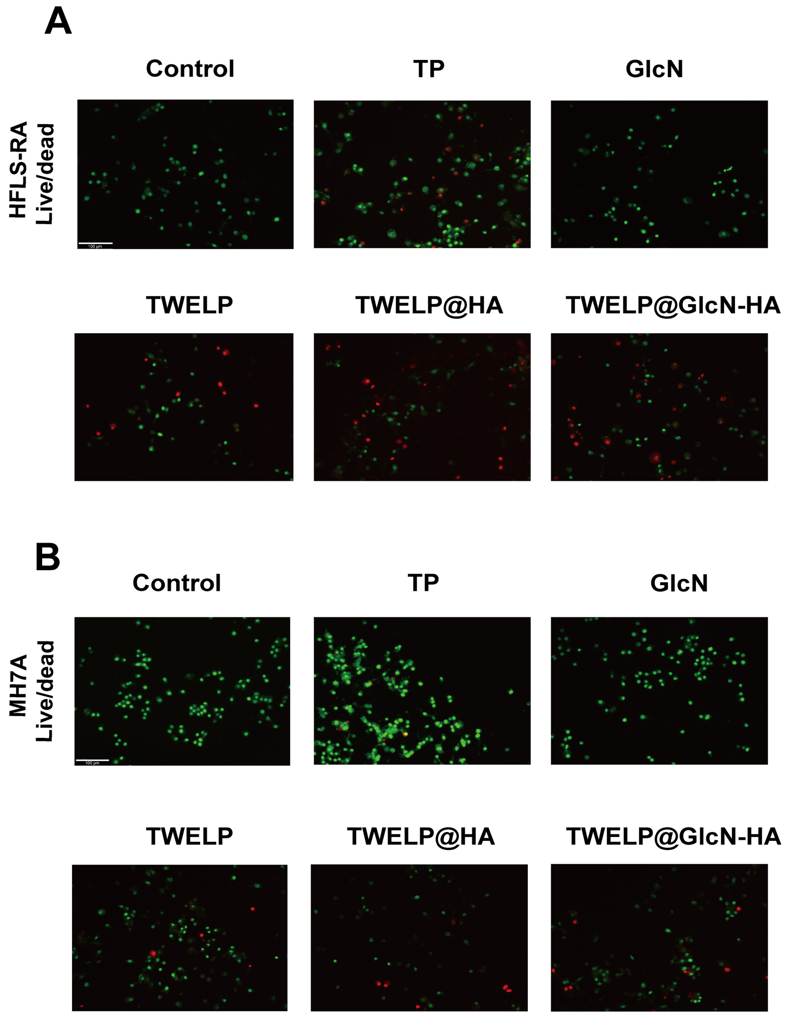

Supplementary Figure 6. (A) Representative Live/Dead staining images of HFLS-RA cells under different treatment conditions. (B) Representative Live/Dead staining images of MH7A cells under different treatment conditions. Scale bar: 100 μm.

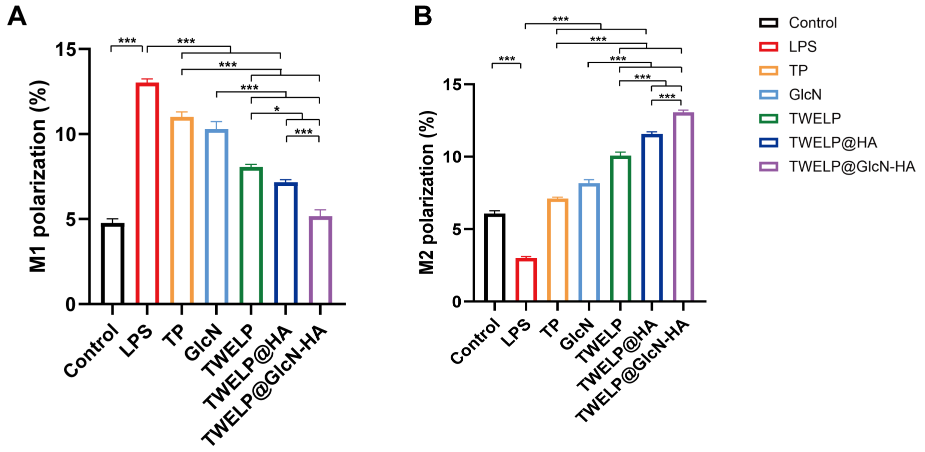

Supplementary Figure 7. Quantitative analysis of macrophage polarization under different treatment conditions. (A) Proportion of CD86-positive macrophages representing M1 polarization. (B) Proportion of CD206-positive macrophages representing M2 polarization. Data are presented as mean ± SD from three independent biological replicates (n = 3). Statistical significance: *$P < 0.05$, **$P < 0.01$, ***$P < 0.001$.

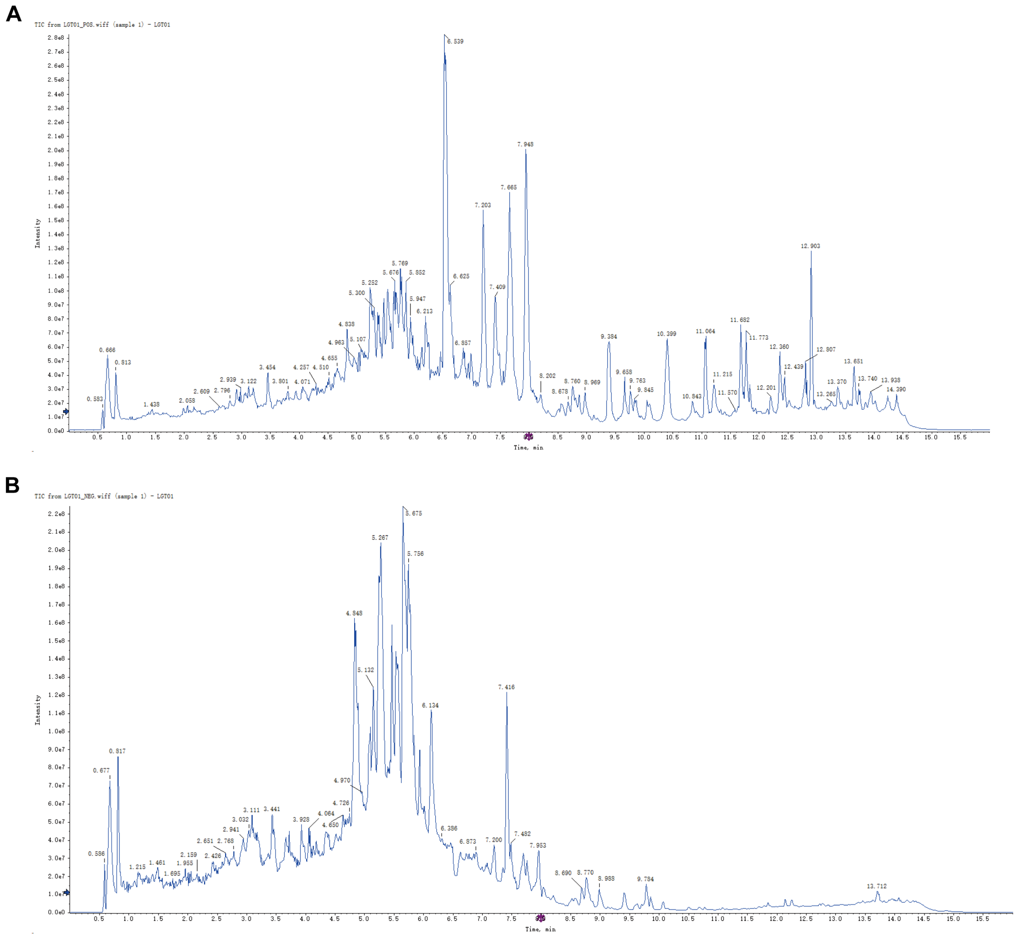

Supplementary Figure 8. Representative total ion chromatograms (TICs) of TWELP analyzed by LC–MS. (A) Positive ion mode (POS) TIC displaying the global ion response and peak distribution of TWELP metabolites across retention time. (B) Negative ion mode (NEG) TIC showing the overall metabolic profile and signal intensity

variation throughout the chromatographic separation.

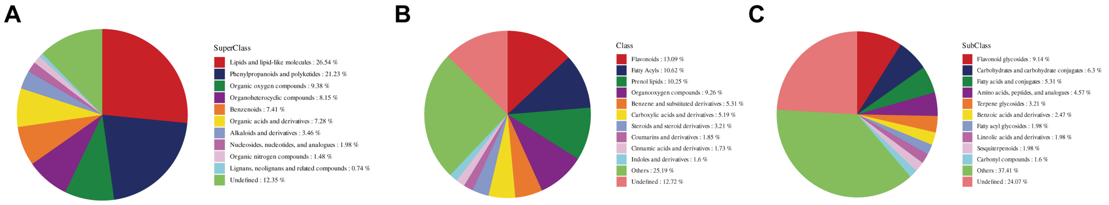

Supplementary Figure 9. (A) Pie chart of chemical classification of identified metabolites at the SuperClass level, illustrating the proportional distribution of different metabolite categories. (B) Pie chart of chemical classification of identified metabolites at the Class level, showing the proportional distribution of major metabolite types. (C) Pie chart of chemical classification of identified metabolites at the SubClass level, presenting the compositional proportions of specific metabolite subclasses.

3. ^{1}H and ^{13}C NMR Spectrum of key Compounds

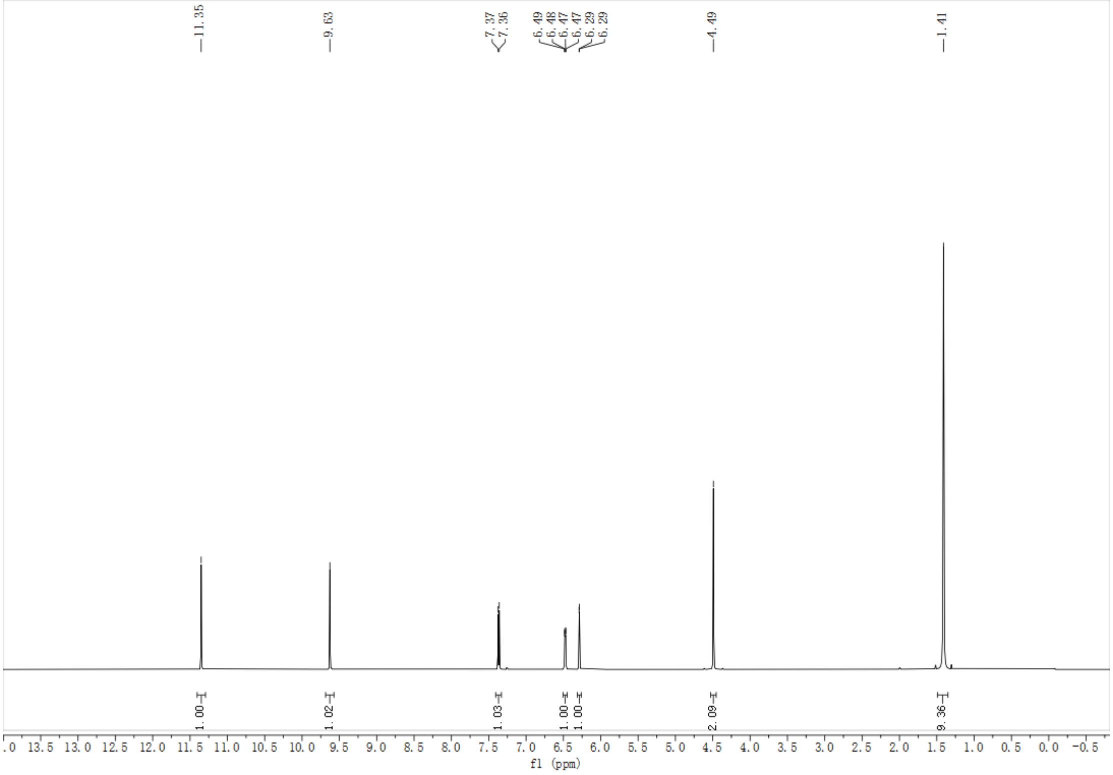

^{1}H NMR spectrum of **2** in Chloroform-*d* (600 MHz)

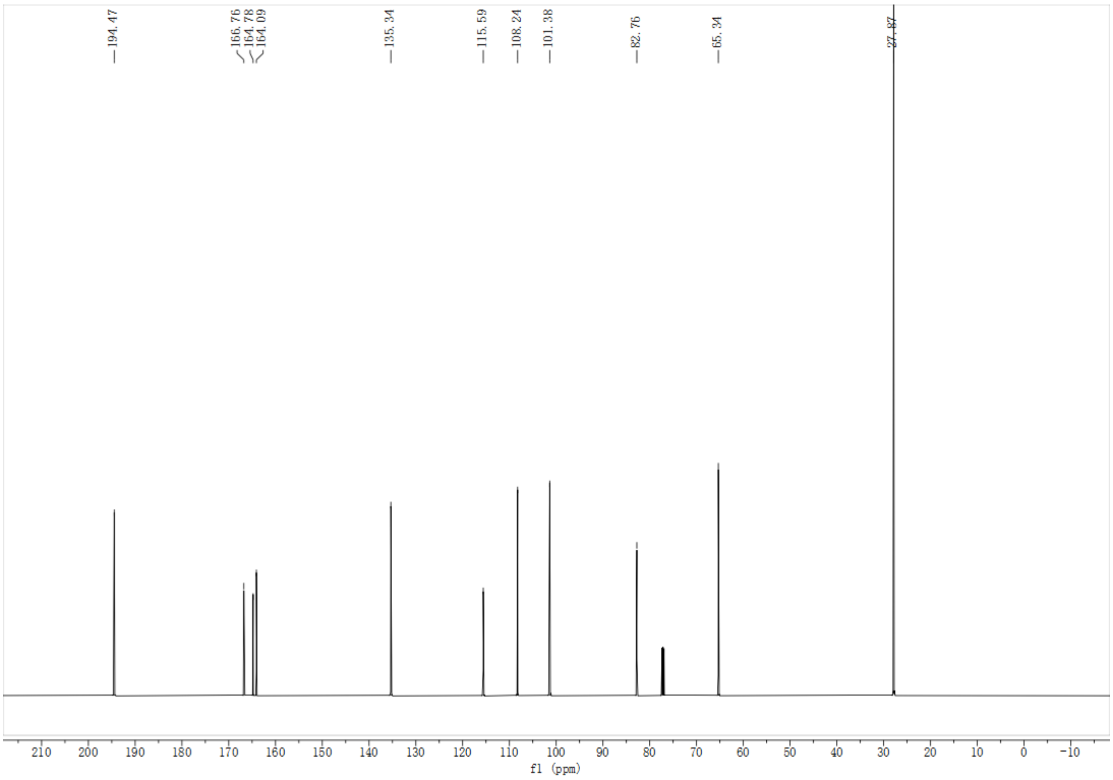

^{13}C NMR spectrum of **2** in Chloroform-*d* (150 MHz)

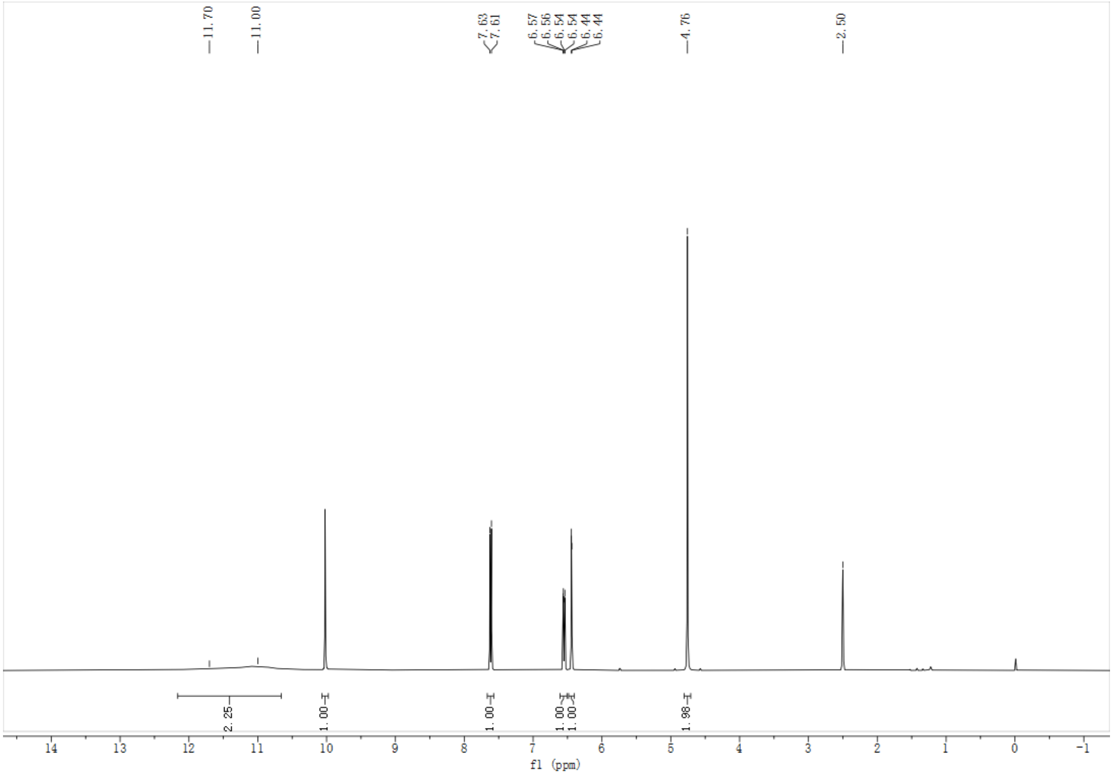

^{1}H NMR spectrum of **3** in DMSO-d_6 (400 MHz)

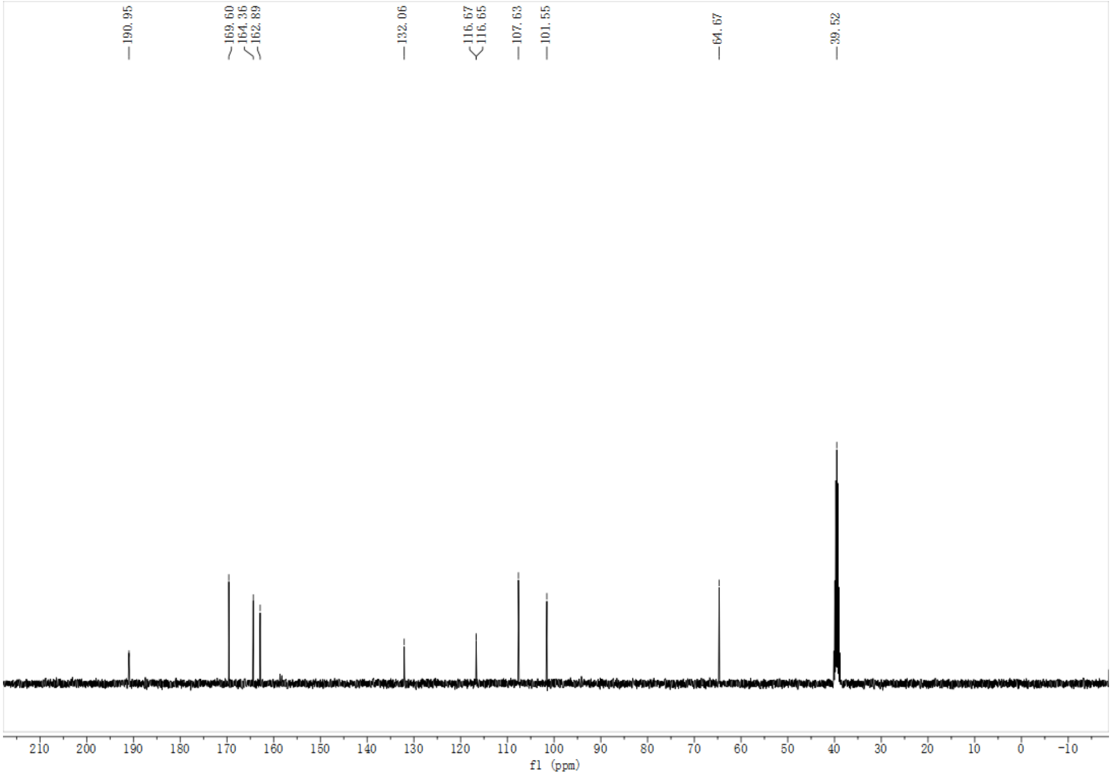

^{13}C NMR spectrum of **3** in DMSO-d_6 (100 MHz)

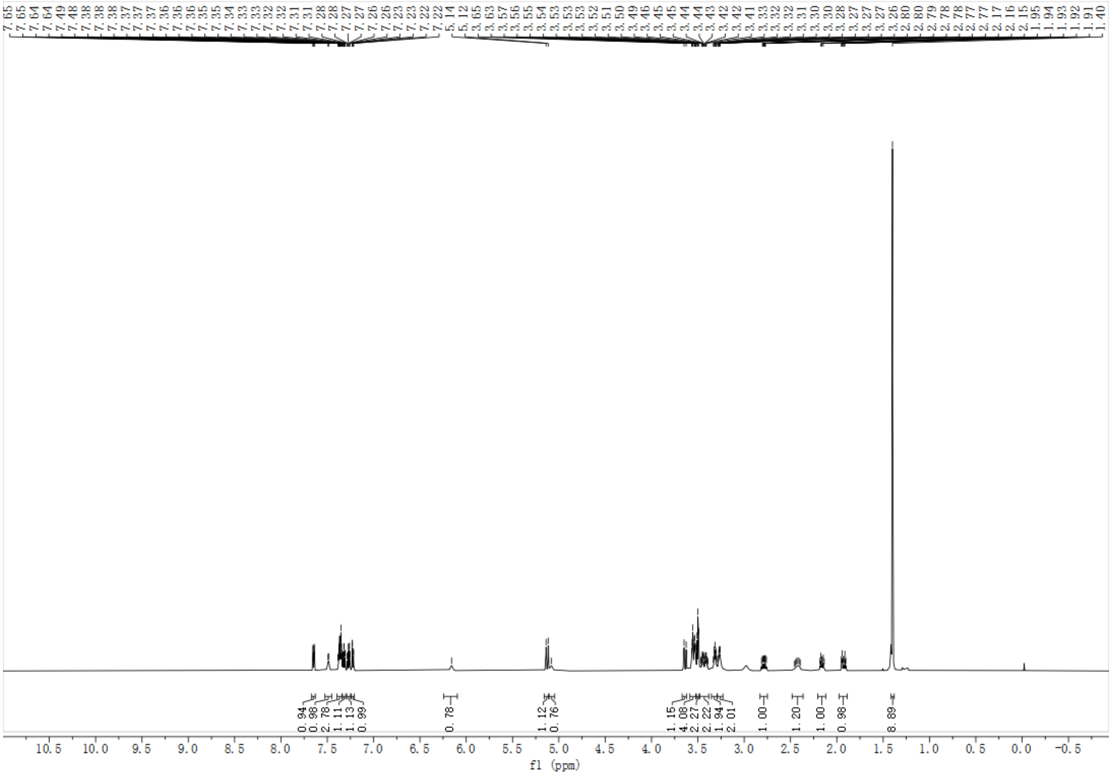

^{1}H NMR spectrum of **6** in Chloroform-*d* (600 MHz)

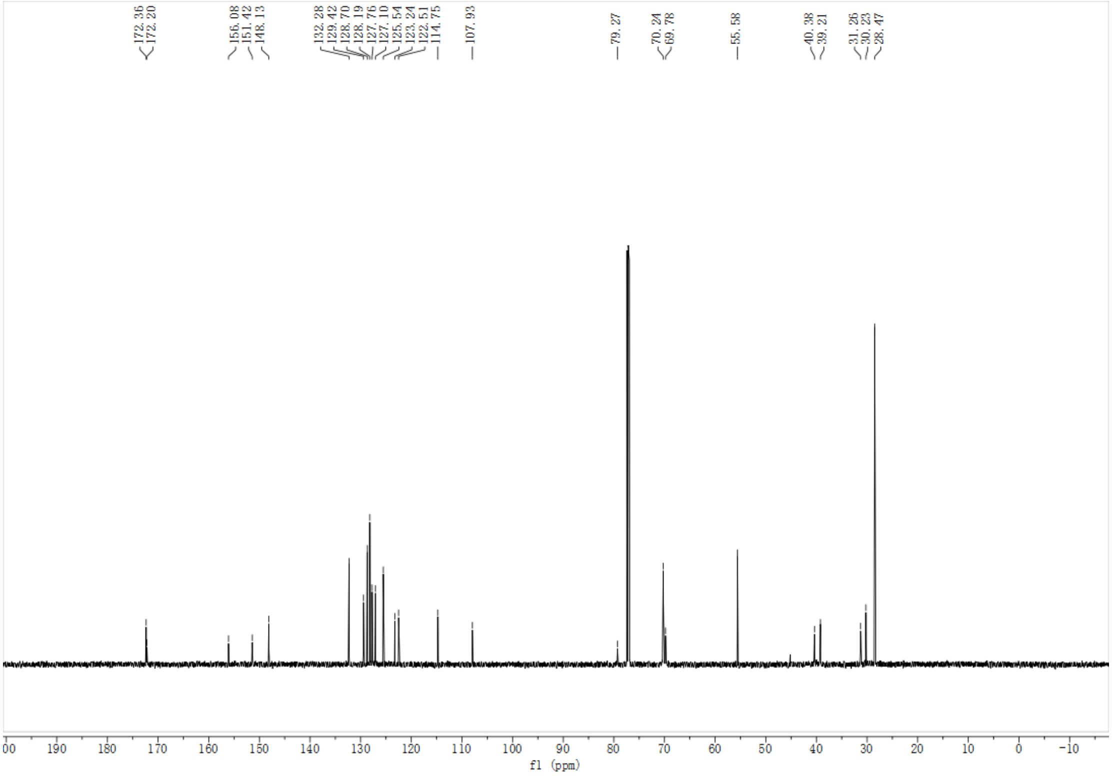

^{13}C NMR spectrum of **6** in Chloroform-*d* (150 MHz)

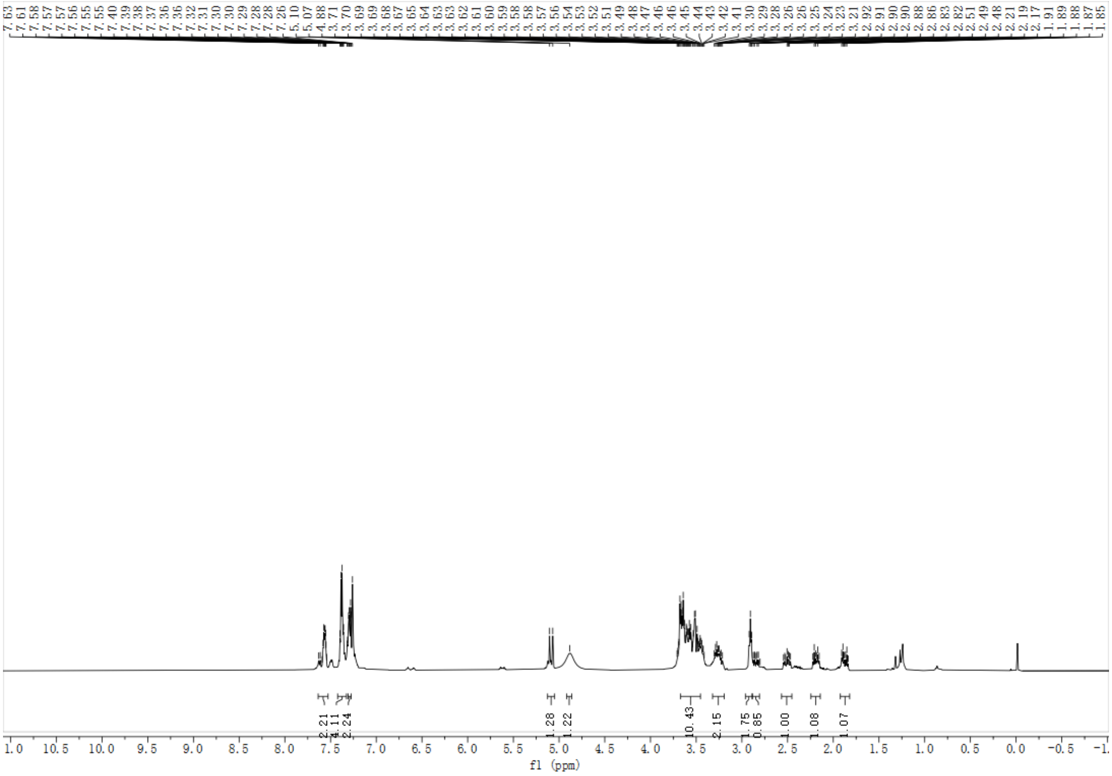

^{1}H NMR spectrum of **7** in Chloroform-*d* (400 MHz)

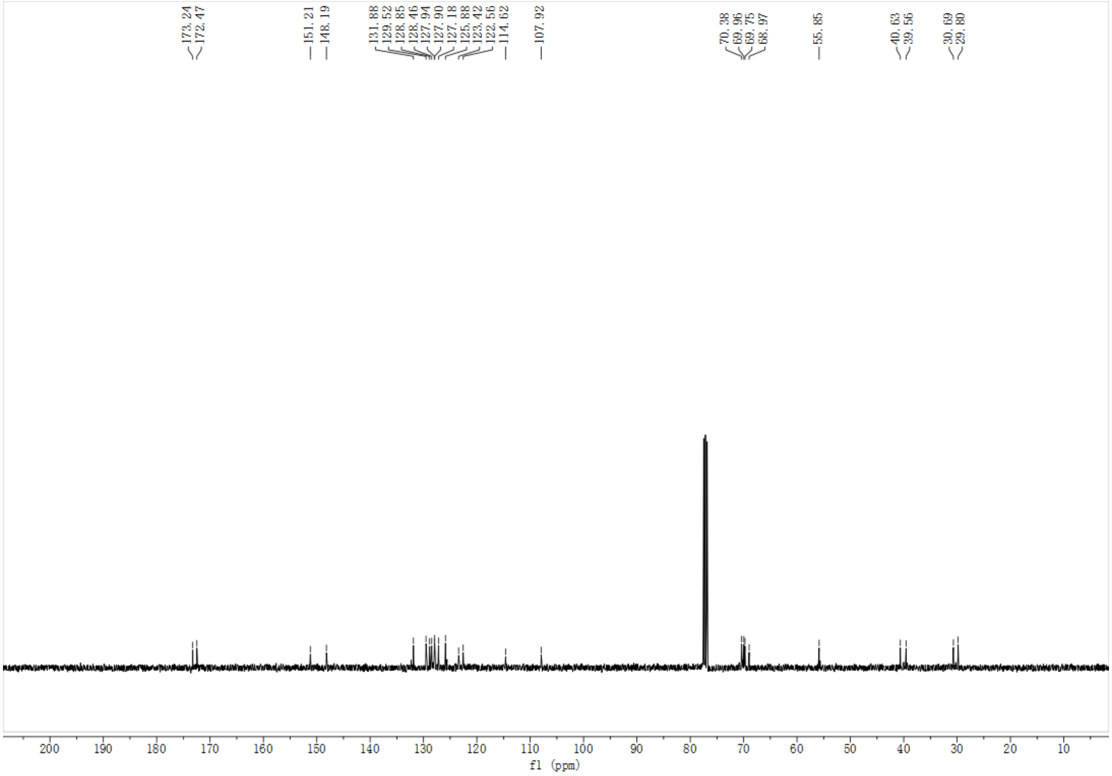

^{13}C NMR spectrum of **7** in Chloroform-*d* (100 MHz)

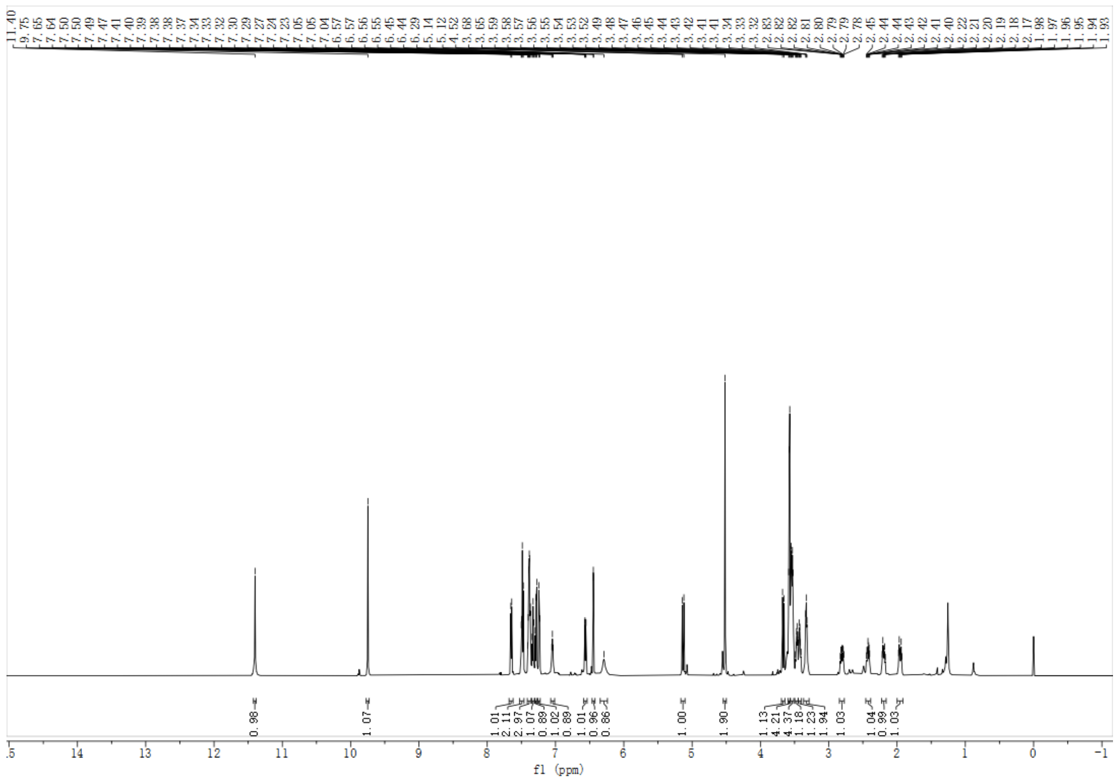

^{1}H NMR spectrum of **8** in Chloroform-*d* (600 MHz)

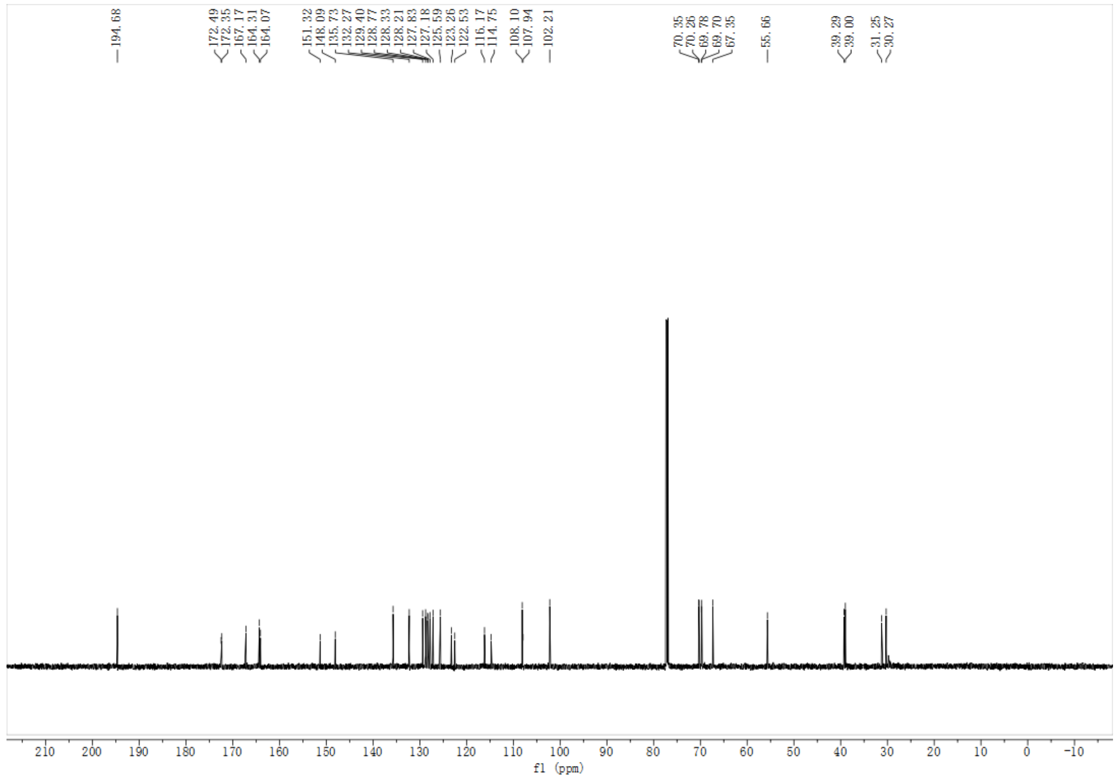

^{13}C NMR spectrum of **8** in Chloroform-*d* (150 MHz)

References

[1] D. Li, S. Yao, Z. Zhou, J. Shi, Z. Huang, Z. Wu, Hyaluronan decoration of milk exosomes directs tumor-specific delivery of doxorubicin, Carbohydr Res, 493 (2020) 108032.
[2] C.E. Lee, S. Kim, H.W. Park, W. Lee, A.K. Jangid, Y. Choi, W.J. Jeong, K. Kim, Tailoring tumor-recognizable hyaluronic acid-lipid conjugates to enhance anticancer efficacies of surface-engineered natural killer cells, Nano Converg, 10 (2023) 56.
